# High Temperature Induces Expression of Tobacco Transcription Factor NtMYC2a to Regulate Nicotine and JA Biosynthesis

**DOI:** 10.3389/fphys.2016.00465

**Published:** 2016-10-27

**Authors:** Liming Yang, Junying Li, Jianhui Ji, Ping Li, Liangliang Yu, Elsayed F. Abd_Allah, Yuming Luo, Liwei Hu, Xiangyang Hu

**Affiliations:** ^1^Jiangsu Collaborative Innovation Center of Regional Modern Agriculture & Environment Protection, Jiangsu Key Laboratory for Eco-Agriculture Biotechnology around Hongze Lake, Huaiyin Normal UniversityHuaian, China; ^2^Department of Plant Pathology, University of GeorgiaTifton, GA, USA; ^3^Department of Agronomy, Yunnan Academy of Tobacco Agricultural SciencesKunming, China; ^4^Shanghai Key Laboratory of Bio-Energy Crops, School of Life Sciences, Shanghai UniversityShanghai, China; ^5^Plant Production Department, College of Food and Agricultural Sciences, King Saud UniversityRiyadh, Saudi Arabia; ^6^Seed Pathology Department, Plant Pathology Research Institute, Agriculture Research CenterGiza, Egypt; ^7^Laboratory of Tobacco Agriculture, Zhengzhou Tobacco Research Institute of CNTCZhengzhou, China

**Keywords:** NtMYC2a gene, high temperature stress, nicotine biosynthesis, Jasmonic acid, expression regulation, protein interactions

## Abstract

Environmental stress elevates the level of jasmonic acid (JA) and activates the biosynthesis of nicotine and related pyridine alkaloids in tobacco (*Nicotiana tabacum* L.) by up-regulating the expression of putrescine *N*-methyltransferase 1 (NtPMT1), which encodes a putrescine *N*-methyl transferase that catalyzes nicotine formation. The JA signal suppressor JASMONATE ZIM DOMAIN 1 (NtJAZ1) and its target protein, NtMYC2a, also regulate nicotine biosynthesis; however, how these proteins interact to regulate abiotic-induced nicotine biosynthesis is poorly understood. In this study, we found that high-temperature (HT) treatment activated transcription of *NtMYC2a*, which subsequently stimulated the transcription of genes associated with JA biosynthesis, including *Lipoxygenase* (*LOX*), *Allene oxide synthase* (*AOS*), *Allene oxide cyclase* (*AOC*), and *12-oxophytodienodate reductase* (*OPR*). Overexpression of *NtMYC2a* increased nicotine biosynthesis by enhancing its binding to the promoter of *NtPMT1*. Overexpression of either *NtJAZ1* or proteasome-resistant *NtJAZ1*Δ*C* suppressed nicotine production under normal conditions, but overexpression only of the former resulted in low levels of nicotine under HT treatment. These data suggest that HT induces NtMYC2a accumulation through increased transcription to activate nicotine synthesis; meanwhile, HT-induced NtMYC2a can activate JA synthesis to promote additional NtMYC2a activity by degrading NtJAZ1 at the post-transcriptional level.

## Introduction

Tobacco (*Nicotiana tabacum*) generates an array of alkaloids that play essential roles in the plant defense response against herbivore and insect attack (Kessler and Baldwin, 2002; Steppuhn et al., 2004). Nicotine is the main alkaloid produced by cultivated tobacco (*N. tabacum* L.), constituting ~0.6–3% of the tobacco leaf dry weight. Nicotine is synthesized in the root from ornithine and arginine by way of putrescine. Putrescine is either metabolized to higher polyamines, such as spermidine and spermine, or conjugated with cinnamic acid derivatives or fatty acids in all higher plants; however, it is also converted into *N*-methylputrescine in plants that produce nicotine or tropane alkaloids. Thus, putrescine *N*-methyltransferase (PMT; EC 2.1.1.53) participates in the first committed step of alkaloid biosynthesis (Chattopadhyay and Ghosh, 1998; Chou and Kutchan, 1998). *N*-Methylputrescine is then oxidized by a diamine oxidase (EC1.4.3.6) and cyclized spontaneously to the l-methyl-A'-pyrrolinium cation, which is condensed with nicotinic acid or its derivative. Quinolinic acid phosphoribosyltransferase (QAPRT; EC2.4.2.19) serves as the entry-point enzyme in the pyridine nucleotide cycle, which supplies nicotinic acid. After biosynthesis in the tobacco root, nicotine is translocated to the leaf via the xylem and stored in the leaf vacuole with the help of a tonoplast-localized transporter. Nicotine can be demethylated in both leaves and roots, but it is primarily demethylated in senescing leaves (Wagner et al., 1986; Chou and Kutchan, 1998). The accumulation of nicotine in tobacco is affected by environmental factors, culture practices, and plant hormone levels. For example, the application of nitrogen fertilizer or jasmonic acid (JA) markedly increases nicotine biosynthesis (De Luca and St Pierre, 2000; Shoji et al., 2000; Facchini, 2001; Goossens et al., 2003; Paschold et al., 2007). However, little is known about the underlying mechanism.

JA and its oxylipin derivatives, collectively called jasmonates, play important roles in multiple physiological processes, including defense responses against abiotic and biotic stresses (Farmer et al., 2003; Robert-Seilaniantz et al., 2011). The jasmonate signal reprograms the transcriptional profiles that determine a set of physiological responses (Chini et al., 2007; Niu and Figueroa, 2011). MYC2, a basic helix-loop-helix (bHLH) transcription factor in *Arabidopsis thaliana*, and its homologs regulate the expression of a broad range of jasmonate-responsive genes by directly binding to their G-boxes (Xu and Timko, 2004; Gangappa and Chattopadhyay, 2013). Recently, the jasmonate signal was also found to regulate another bHLH factor, ICE1, to enhance Arabidopsis tolerance to freezing stress (Hu et al., 2013). The jasmonate signal is involved in the biosynthesis of defense-related secondary metabolites. The perception of a JA signal through JA-Ile by the receptor F-box protein CORONATINE INSENSITIVE1 (COI1) leads to the formation of a stable COI1/JA-Ile complex, which subsequently degrades the JASMONATE ZIM DOMAIN (JAZ) protein family via the 26S proteasome complex (Chung et al., 2008; Sheard et al., 2010; Yan et al., 2013). In the absence of JA-Ile, JAZ proteins bind to their target proteins, such as MYC2a, to inactivate the downstream JA signal. However, once JAZs have been degraded in the presence of JA-Ile, MYC2a is released to activate the downstream components of the JA signaling pathway alone or in cooperation with other transcription factors (Lorenzo et al., 2004; Chini et al., 2007). The function and stability of JAZs are strictly controlled by endogenous hormones and exogenous environmental signals. For example, the gibberellin-responsive DELLA proteins interact with JAZs to promote their degradation in Arabidopsis (Hou et al., 2010; Yang et al., 2012; Qi et al., 2014). Zhang et al. reported that the tobacco transcription factors *NtMYC2a* and *NtMYC2b* form a nuclear complex with NtJAZ1 to regulate jasmonate-induced nicotine biosynthesis (Zhang et al., 2012), suggesting that NtJAZ1 and NtMYC2 interact to control nicotine biosynthesis.

To explore the underlying molecular mechanism whereby environmental factors affect nicotine biosynthesis, we examined the effect of several environmental stress factors, including high temperature (HT, 32°C), wounding, salinity, and heavy metal stress, on nicotine production. We found that HT treatment effectively enhanced nicotine biosynthesis in tobacco. Further analysis demonstrated that HT increased transcription of *NtMYC2a*. On the one hand, HT-induced NtMYC2a can bind the promoter region of *NtPMT1* to induce nicotine synthesis. On the other hand, HT-induced NtMYC2a increased the expression of genes including *NtLOX, NtAOS, NtAOC*, and *NtOPR* and finally induced the accumulation of JA. NtMYC2a-mediated JA accumulation further decreased the stability of NtJAZ1 thus promoted additional NtMYC2a activity for accelerate JA biosynthesis. Based on these findings, we propose that *NtMYC2a* plays the bifunctional roles in HT-induced nicotine biosynthesis at the transcriptional and post-transcriptional level. Overall, our results reveal a novel mechanism that HT induces nicotine biosynthesis by precisely modulating NtMYC2a in tobacco.

## Materials and methods

### Plant materials

Sterilized tobacco (*N. tabacum* cv. Wisconsin 38) seeds were germinated and grown to seedlings under continuous illumination on half-strength Gamborg B5 medium solidified with 2% (w/v) gellan gum and supplemented with 0.3% sucrose at 24°C. Two-week-old plants were transferred to Perlite saturated with half-strength Gamborg B5 medium and grown for another 2 weeks in the greenhouse at 24°C before HT treatment. For HT treatment, the 4-week-old seedlings were placed in a plant growth chamber at 32°C for the indicated time. The plants were placed in a growth chamber at room temperature for the same amount of time as the control. For MeJA treatment, MeJA at different concentrations was sprayed on leaves of the 4-week-old tobacco plants. For saline or heavy-metal treatment, 100 mM NaCl or 30 μM CdCl_2_, respectively, was used to water the 4-week-old tobacco seedlings for the indicated times. For wounding treatment, the leaves were wounded with a pattern wheel. After each treatment, the tobacco roots were immediately collected for further molecular analysis and alkaloid measurement. The roots were frozen immediately in liquid nitrogen for later analysis.

### Alkaloid analysis

A 0.5-g sample from each of the collected tobacco roots was collected and frozen in liquid nitrogen. The frozen samples were lyophilized and then homogenized in 4 ml of 0.1 M H_2_SO_4_. The homogenate was sonicated for 60 min and centrifuged at 2000 g for 15 min. The resulting supernatant was neutralized by adding 0.4 ml 25% NH_4_OH. The mixture was loaded onto an Extrelut-1 column and eluted with 6 ml of chloroform. The eluent was dried at 37°C, and each residue was dissolved in ethanol and analyzed by gas chromatography–mass spectrometry (GC/MS) using a split sampling mode as described in Goossens et al. (2003). The column temperature was held at 100°C for 10 min and then increased to 260°C during a 35-min period, at a gradient of 8°C/min. Signal output was simultaneously monitored for two separate ion pairs for nicotine (m/z 163.2/105.9 and m/z 163.2/80.1) and single ion pairs for anabasine (m/z 162.9/80.1), nornicotine (m/z 149.1/80.1), and nicotine-d3 (m/z 166.3/87.2) during the last 4 min of sample elution. Total elution time monitored was 7.5 min. The stable heavy isotope-labeled Nicotine-d3 (CIJ, MA, US) was used as the internal standard.

### Vector construction and plant transformation

To construct binary vectors overexpressing *NtMYC2a*, total RNA was isolated from tobacco using an RNeasy Mini kit (Qiagen). RNAs were converted to first-strand cDNAs by SuperScriptII Reverse Transcriptase (Invitrogen) with an oligo(dT) primer (Zhang et al., 2012). The coding region of *NtMYC2a* was amplified using the MYC2a-F and MYC2a-R primers. All primer sequences are provided in Supplementary Table 1. The *pOE-6HA* binary vector was derived from *pGREEN* vector with the 35S promoter in the KpnI/XhoI site, and NOS terminator in the NotI/SacI site, and the 6xHA tag fragment were cloned into the XbaI/NotI site. The *pOE-6HA* vector were digested with BamHI/EcoRI, and the *NtMYC2a* fragment was inserted into the BamHI/EcoRI site under the control of the 35S promoter using In-fusion Recombination Enzyme (Clontech) to generate the binary pOE-*NtMYC2a-6HA* construct. The *pRI101-GFP* vector derived from the p RI101-ON binary vector (Clonetech), and GFP fragment were inserted into the NdeI/EcoRI site. Similarly, the DNA fragment of *NtJAZ1* was amplified using the corresponding primers (Supplementary Table 1) and inserted into the EcoRI/HindIII site of the modified *pRI101-GFP* vector under the control of 35S to generate the *pRI101-NtJAZ1-GFP* binary construct. To generate the *NtJAZ1*Δ*C* fragment, which lacks the JAS domain (containing PIARRNSLTRFLEKRKDRITSTAPYQI) between 181 and 207 site of NtJAZ1, overlapping primers that span the JAS domain were used (see Supplementary Table 1 for the detail primer sequences) to amplify the *NtJAZ1* template to produce *NtJAZ1*Δ*C* using a QuickChange Site-Directed Mutagenesis kit (Catalog #200521, Stratagene). The *NtJAZ1*Δ*C* fragment was then inserted into the EcoRI/HindIII site of the modified pRI101-GFP vector using In-Fusion recombination enzyme (Clontech) to generate the binary pRI101-NtJAZ1ΔC-GFP construct. The constructed binary vector was then transformed into the *Agrobacterium tumefaciens* strain GV3101. To generate transgenic tobacco, tobacco leaf discs were infected with *A. tumefaciens* strain GV3101 harboring a binary vector, following a previous method (An, 1985), and the transgenic tobacco seedlings were screened on the selective medium containing 100 mg/L kanamycin sulfate), shoots that were ≥5 cm high were transferred to peat compost and grown to maturity in the greenhouse. Primary transformation lines were propagated up to the T_2_ generation by self-pollination.

### Protoplast transient expression assays

The pGREENII0800-LUC vector were derived from pGREEN vector, and is widely used in the analysis of promoter transcription factor interactions, such vector contained the LUC reporter marker in the NcoI/XbaI site, and a REN gene under the control of a 35S promoter in the BglI/KpnI site for estimating the extent of transient expression (Hellens et al., 2005). To perform a transient transcriptional activity assay using the luciferase reporter, the 755-bp *NtPMT1a* promoter was amplified from genomic DNA and inserted into pGREENII0800-LUC to generate the *pNtPMT1a: LUC* reporter construct. To generate the mutated *pNtPMT1a: LUC* reporter construct, the mutated site in the promoter region of *NtPMT1a* was generated using the QuickChange Site-Directed Mutagenesis kit. The coding region of *NtMYC2a* was amplified and inserted into the pGREEN62-SK vector under the control of the 35S promoter to generate the effector construct. Root tissue was collected from 4-week-old tobacco plants. Root cell protoplasts were isolated, and polyethylene glycol (PEG)–mediated transient transformation was performed as described (Sheen, 2001). In brief, for each transformation, 10 μg reporter construct and 10 μg effector construct were introduced into 200 μL of protoplast solution (~2 × 10^6^ cells). Transformed protoplasts were resuspended in 4 ml liquid MS medium with 0.4 M sucrose and incubated at 24°C in darkness overnight or subjected to heat shock at 32°C for 30 min and then incubated at 24°C in darkness overnight. We firstly obtained the protoplast coexpressing the *pNtPMT1a: LUC* reporter construct and *NtMYC2a* or empty effector constructs by PEG-mediated transformation, and then co-transformed the *JAZ1* or *JAZ1*Δ*C* effector construct into the protoplast with *pNtPMT1a: LUC/NtMYC2a* to obtained the protoplast with triple constructs (*pNtPMT1a: LUC/NtMYC2a/JAZ1 or pNtPMT1a: LUC/NtMYC2a/JAZ1*Δ*C*). For the luciferase activity assay, samples were lysed in a buffer containing 100 mM potassium phosphate (pH 7.5) and 1 mM DTT and were cleared by centrifugation at 12,000 g for 5 min. Then, 15 μL of sample was used to measure the luciferase and Renilla luciferase (REN) activities with the dual-luciferase reporter assay system (Promega). Data are presented as the ratio of luciferase activity relative to the control REN activity.

### Protein isolation and immunoblotting analysis

Proteins were isolated with extraction buffer containing 50 mM Tris-HCl, pH 7.5, 150 mM NaCl, 1 mM PMSF, 1 × Complete Protease Inhibitor Cocktail (Roche), 5% glycerol, 1 mM EDTA, and 1 mM DTT. The protein concentration was determined with the Bradford assay (Bio-Rad). The samples were mixed with 2 × SDS sample buffer and boiled for 3 min and then separated on a 10% SDS-polyacrylamide gel. The membrane transfer and protein gel blot assays were performed as described (Wang et al., 2009; Zhang et al., 2013). Subsequent immunoblotting assays were performed with anti-GFP (Clontech) and anti-Actin (Agrisera) at dilutions of 1:3000 and 1:2000, respectively.

### Chromatin immunoprecipitation (ChIP)

ChIP experiments were performed as described (Ouyang et al., 2011) using root tissue from 4-week-old transgenic seedlings that had been subjected to 24 h of HT treatment at 32°C or to control conditions. In brief, 5 g of root tissue was cross-linked in 1X PBS buffer (25 mL) with 1% formaldehyde on ice under vacuum; the vacuum was released after 15 min and reapplied for another 14 min. Fixation was stopped by adding glycine to a final concentration of 0.125 M, and the fixed sample was washed three times with MC buffer and ground to a powder in liquid nitrogen, followed by nuclear isolation by adding the frozen powder to 30 mL of M1 buffer. The homogenate was filtered through four layers of Miracloth prior to nuclei isolation. Nuclear-enriched extracts were resuspended in 5 mL lysis buffer (50 mM HEPES, pH 7.5, 150 mM NaCl, 1 mM EDTA, 1% Triton X-100, 0.1% deoxycholate, 0.1% SDS, 1 mM phenylmethylsulfonyl fluoride, and 10 mM sodium butyrate) containing a plant proteinase inhibitor cocktail (Sigma-Aldrich), followed by sonication for 5 min on medium power in 1.5 mL sonic buffer using a Bioruptor UCD-200 (Diangenode) and centrifugation at 500 g to remove starch granules. The chromatin solution was sonicated for 5 min on medium power five times to create ~300-bp average fragment sizes, as estimated by agarose gel electrophoresis. Antibody against HA and the IgG (Sigma-Aldrich) control were used for immunoprecipitation. The precipitated DNA was recovered using a QIAquick PCR purification kit (Qiagen) and analyzed by real-time quantitative PCR using the appropriate DNA primers (Supplementary Table 1) and SYBR Green Real-Time PCR Master Mix (Applied Biosystems).

### Confocal microscopy

The transgenic NtJAZ1–green fluorescent protein (GFP) plants were treated with or without HT for the indicated periods, and the GFP fluorescence in the root tip was observed under a confocal laser scanning microscope (Olympus). GFP excitation was produced with the 488-nm line of an argon ion laser, and emission was detected between 505 and 530 nm (Tsien and Waggoner, 1995).

### Determination of JA and JA-Ile amounts

JA and JA-Ile were quantified using a high-performance liquid chromatography (HPLC)-MS/MS approach as reported (Vadassery et al., 2012). Frozen leaf tissue (~200 mg) was ground in liquid nitrogen. Phytohormones were extracted with 1 ml of ethyl acetate spiked with internal standards (100 ng of [^2^H_6_]-JA and 20 ng each of JA-[^13^C_6_]-Ile, [^2^H_6_]-JA and JA-[^13^C_6_]-Ile purchased from OlChemIm Ltd, Czech Republic). After extraction by vortexing for 10 min, the organic phase was obtained by centrifugation at 1600 g for 15 min at 4°C. Samples were evaporated almost to dryness in a vacuum concentrator (Eppendorf, Hamburg, Germany) under reduced pressure at 30°C. Leaf samples were then diluted in 600 μl of 70:30 (v/v) methanol/water. Analysis was performed with a Shimadzu 8040 HPLC-MS/MS system. Chromatography was performed on a Shimadzu FPLC system (Shimadzu). Separation was achieved on a Shimadzu pack XR-ODS column (2.0 mm i.d., 75 mm; Shimadzu). Mobile phases consisted of 0.05% [(v/v)] formic acid and 5 mM ammonium formate in water (A) and methanol (B). The elution profile was as follows: 0–0.01 min, 20% B; 0.01–8.0 min, 20–95% B; 8.0–8.1 min, 20% B. The mobile phase flow rate was 0.3 mL min^−1^. The column temperature was maintained at 50°C. An ESI tandem mass spectrometer (Shimadzu) was operated in the negative ionization mode. The instrument parameters were optimized by infusion experiments with pure standards, where available. Molecular ions [M-H]^−^ at mass-to-charge ratio (m/z) 209.1 and 322.2, generated from endogenous JA and JA-Ile, were fragmented. Multiple reaction monitoring was used to monitor analyte parent ion → product ion: Mass-to-charge ratio m/z 209.1 → 59.0 (CE, −14 V; Q1 pre bias, −12V; Q3 pre bias, −22V) for JA; m/z 215.1 → 62.0 (CE, −10 V; Q1 pre bias, −12V; Q3 pre bias, −24V) for [^2^H_6_]-JA; m/z 322.2 → 130.1 (CE, −21 V; Q1 pre bias, −21V; Q3 pre bias, −23V) for the JA-Ile conjugate; and m/z 328.2 → 130.1 (CE, −22 V; Q1 pre bias, −25V; Q3 pre bias, −23V) for the JA-[^13^C_6_]Ile conjugate.

## Results

### HT induces the accumulation of nicotine and up-regulates the transcription of *NtMYC2a*

**Figure 1:**
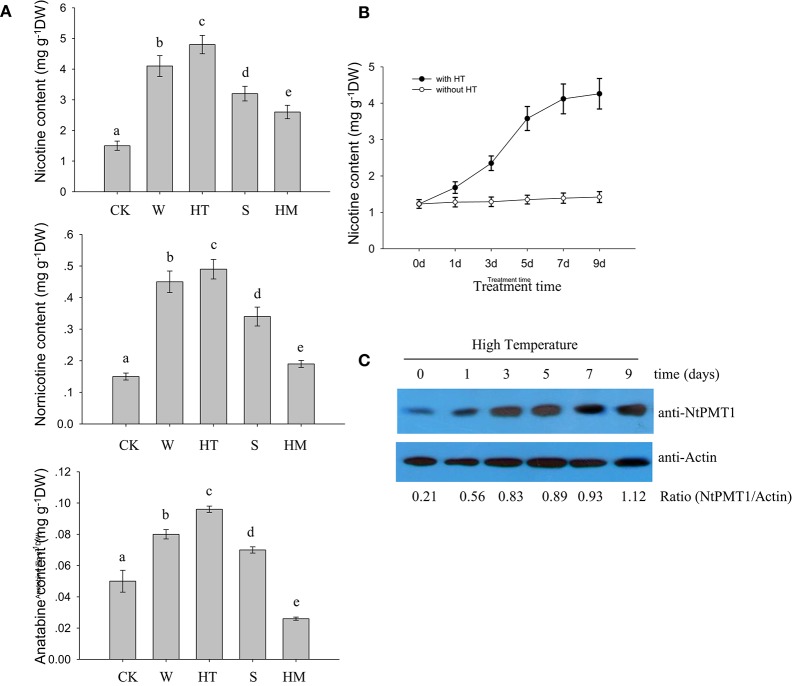
**HT induces ***NtPMT1*** accumulation and nicotine biosynthesis**. Data are the mean ± *SD* from triplicate experiments. Bars with different letters are significantly difference at *p* < 0.05 (Tukey's test). **(A)** The effects of different treatments on alkaloid content in the roots of tobacco seedlings. Four-week-old tobacco seedlings were exposed to leaf wounding (W), HT at 32°C, salinity (S) at 100 mM NaCl, or heavy metal (HM) at 30 μM CdCl_2_ for 3 days or under normal condition at 24°C as the control (CK), and the contents of the indicated alkaloids were measured. **(B)** Time course showing the effect of HT on the induction of nicotine biosynthesis in the roots of tobacco seedlings. The 4-week-old seedlings were grown at 32°C for the indicated periods of time, and the nicotine content was measured. **(C)** HT induces NtPMT1 accumulation. Four-week-old seedlings were grown at 32°C for the indicated time, and the root tissue was used for the analysis. NtPMT1 accumulation was monitored by immunoblotting using anti-NtPMT1. Anti-Actin was used as a loading control.

To investigate the effects of different environmental factors on nicotine biosynthesis in roots from 4-week-old hydroponic tobacco seedlings, we monitored the effects of various stresses, such as HT, wounding, salinity, and heavy metal cadmium treatment, on different alkaloid content, including nicotine, nornicotine, and anatabine. We found that HT, wounding and salinity treatments increased the contents of nicotine, nornicotine in tobacco roots, but cadmium treatment only increased the contents of nicotine and nornicotine, but not anatabine (Figure 1A). Among these treatments, HT treatment resulted in the greatest increase in these alkaloid content (Figure 1A). In a time course experiment, HT treatment caused a gradual increase in nicotine, nornicotine, and anatabine, which still sustained high level after 9 days of treatment (Figure 1B). Given that NtPMT1 is the key enzyme in the nicotine biosynthesis pathway (Shoji and Hashimoto, 2011; Zhang et al., 2012), we evaluated the effect of HT on NtPMT1 accumulation in tobacco roots using NtPMT1 antibody. We found that the level of NtPMT1 increased after 1 day of HT treatment and remained high for at least 9 days of HT treatment (Figure 1C).

### HT induces *NtMYC2a* transcripts and activates the JA synthesis pathway

**Figure 2:**
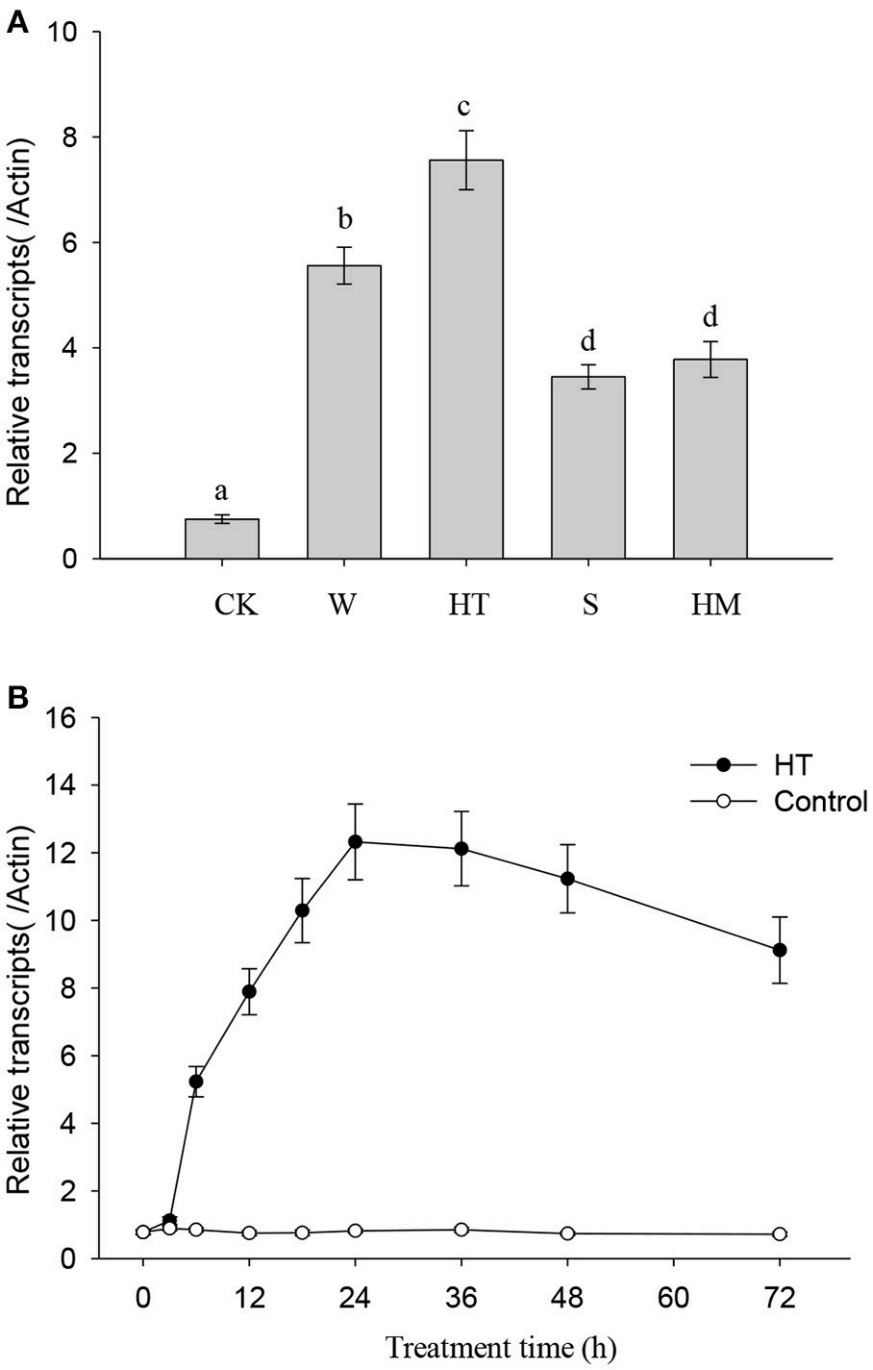
**HT induces ***NtMYC2a*** transcripts**. Data are the mean ± *SD* from triplicate experiments. Bars with different letters are significantly difference at *p* < 0.05 (Tukey's test). **(A)** The effects of different treatments on *NtMYC2a* expression in the roots of tobacco seedlings. Four-week-old tobacco seedlings were exposed to leaf wounding, HT, salinity at 100 mM NaCl, heavy metal at 30 μM CdCl_2_, or under normal condition at 24°C as the control (CK), for 24 h, and transcripts of *NtMYC2a* were measured. **(B)** Time course showing the effect of HT on the transcriptional level of *NtMYC2a* in the roots of tobacco seedlings. Four-week-old tobacco seedlings were grown at 32°C for the indicated periods of time, and the transcriptional level of *NtMYC2a* was measured by quantitative RT-PCR.

NtMYC2a acts as the key transcriptional factor to regulate nicotine synthesis in tobacco (Zhang et al., 2012). To understand the role of NtMYC2a in nicotine synthesis under different stresses, we determined the transcriptional level of *NtMYC2a* after 12 h of stress treatment. As shown in Figure 2A, we found that all of these treatments, including wounding, HT, and saline or heavy metal exposure, could increase the transcriptional level of *NtMYC2a* in tobacco root tissue. Among these treatments, the effect of HT was substantially higher. We also measured the time course effect of HT treatment on the transcription of *NtMYC2a* in tobacco root tissue. HT treatment activated expression of *NtMYC2a* after 6 h and had a maximal effect after 24 h (Figure 2B). The sustained activation of *NtMYC2a* transcripts even after 72 h of HT treatment (Figure 2B) suggested that NtMYC2a may play a role in HT-induced nicotine biosynthesis.

**Figure 3:**
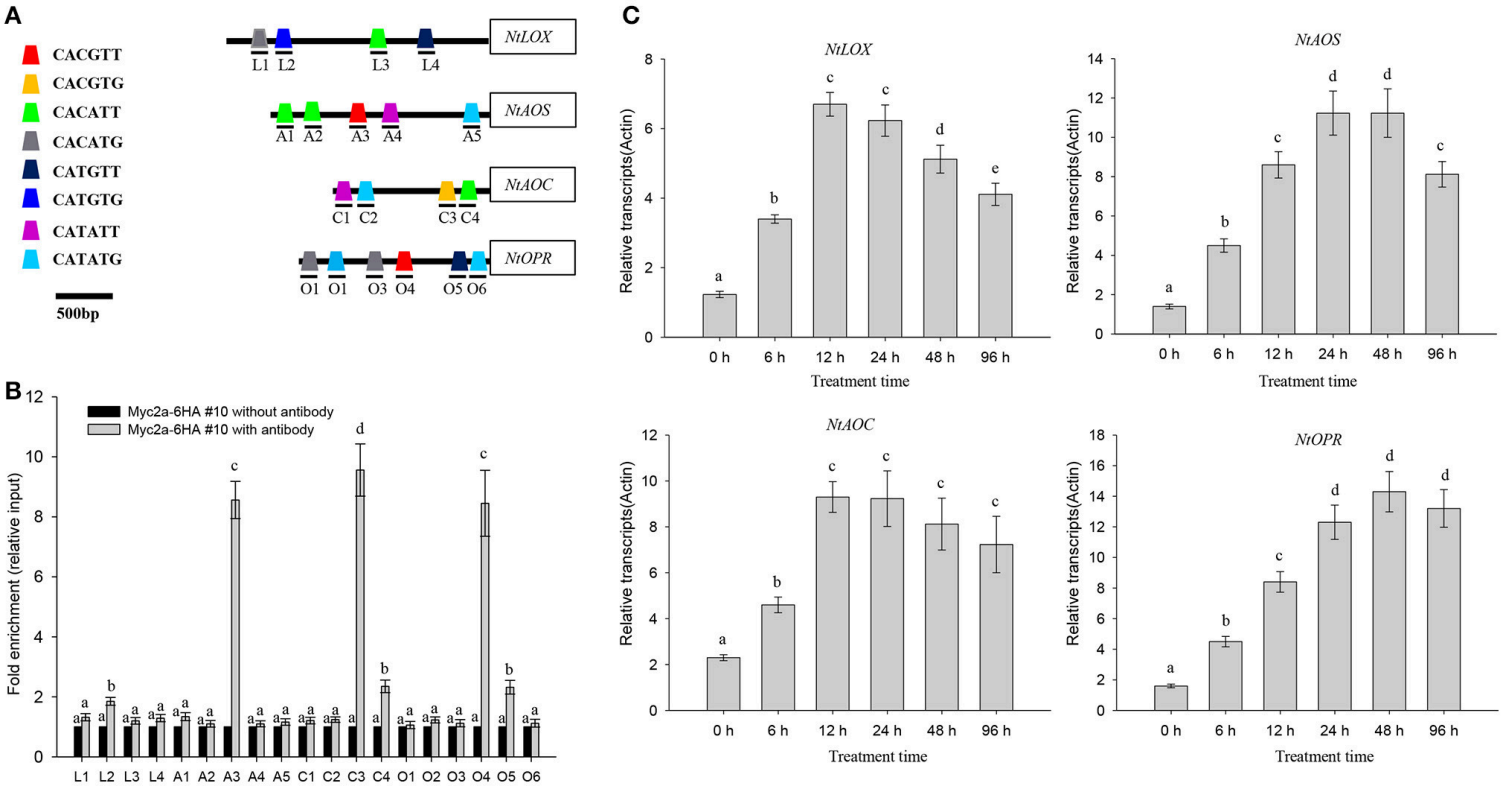
**HT induces transcription of ***NtLOX***, ***NtAOS***, ***NtAOC***, and ***NtOPR***. (A)** NtMYC2a binding sites in the promoter regions of *NtLOX, NtAOS, NtAOC*, and *NtOPR*. L1–4, A1–5, C1–4, and O1–6 denoted different G-box, numbered from the left to right with sequences sites relative to the start code. The box with different color represented the different G-box containing different sequences. **(B)** Quantitative PCR data from the CHIP assay to check the *in vivo* binding ability of NtMYC2a transcriptional factor to the G-box region in the promoters of *NtLOX, NtAOS, NtAOC*, and *NtOPR*. Transgenic *NtMYC2a-GFP #10* plants were used for the assay, ChIP results were normalized to input chromatin, and a fragment in the *ACTIN2* promoter was used as the negative control. L1–4, A1–5, C1–4, and O1–6 means the region as above used for CHIP analysis, the detail information for prime information see Supplementary Table 1. Data are the mean ± *SD* from triplicate experiments. **(C)** HT treatment increases the transcriptional levels of *NtLOX, NtAOS, NtAOC*, and *NtOPR*. Four-week-old tobacco seedlings were exposed to 32°C for indicated time, and the transcriptional levels of *NtLOX, NtAOS, NtAOC*, and *NtOPR* in the plant roots were measured by quantitative qPCR analysis. Data are the mean ± *SD* from triplicate experiments. Bars with different letters are significantly difference at *p* < 0.05 (Tukey's test).

It is reported that NtMYC2a binds to the core G-box motif [5′-CACGT(T/G)-3′] as well as to the G-box-related motif sequences [CA(C/T)(G/A)TT or CA(C/T)(G/A)TG] (Shoji and Hashimoto, 2011). By searching the promoter region, we found a series of NtMYC2a-recognized G-box or G-box-related motifs in promoters of genes associated with JA biosynthesis, including *NtLOX, NtAOS, NtAOC*, and *NtOPR* (Figure 3A; see Supplementary File 1 for detailed information). These results hint that NtMYC2a may bind these motif to activate their expression. To further investigate the role of NtMYC2a in regulating the transcription of JA biosynthesis genes, we generated transgenic tobacco expressing 6HA-fused *NtMYC2a* driven by a 35S promoter (*NtMYC2a-6HA* lines). Quantitative RT-PCR analysis showed that three transgenic tobacco lines all expressed high levels of *NtMYC2a* compared with *Actin* as the control (Supplementary Figure 1A). HA immunoblots of these lines showed that this protein was well expressed within root tissues (Supplementary Figure 1B). Based on complementary ChIP-PCR studies, NTMYC2a strongly interacted with selected components of *NtAOS, NtAOC*, and *NtOPR* promoters, such as the G-motif 5′-CACGT(T/G)-3′, but showed less interaction with fragments with other G-motifs, such as CATAT(T/G) in the promoter of *NtLOX* (Figure 3B). To test this possibility, we measured changes in NtMYC2a-induced gene expression in tobacco root tissue over a time course of HT treatment. Indeed the expression of these four genes was increased after 6 h of HT treatment, and these levels were sustained for the entire 96 h of HT treatment (Figure 3C). Mean wile, we found the higher transcriptional levels of *NtLOX, NtAOS, NtAOC*, and *NtOPR* in the transgenic *NtMYC2a-6HA* lines compared those in the control wild type lines (Supplementary Figure 2). These data are consistent with a previous study (Shoji and Hashimoto, 2011) showing that NtMYC2a binds to different G-motifs with varying affinities and indicate that the G-motif may be important for the binding of NtMYC2a to regulate the expression of JA synthesis–related genes under HT stress.

**Figure 4:**
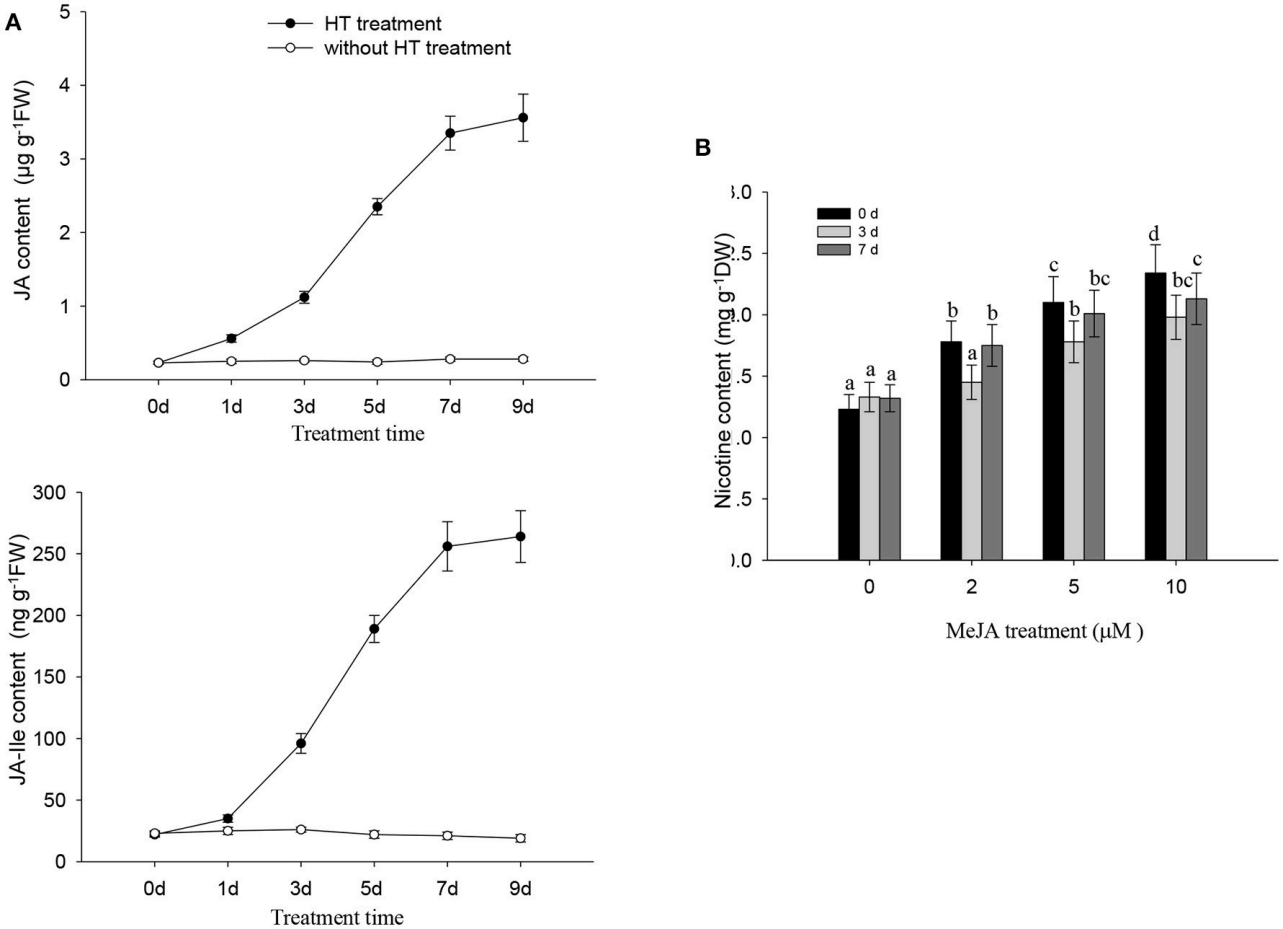
**JA and nicotine biosynthesis are induced by HT and MeJA treatment, respectively**. **(A)** HT induces endogenous production of JA and JA-Ile. Four-week-old plants were grown under HT at 32°C or kept normal condition at 24°C for the indicated number of days. Endogenous JA and JA-Ile levels were measured at various time points during HT treatment. FW, fresh weight. Data are the mean ± *SD* from triplicate experiments. **(B)** the accumulation of nicotine biosynthesis in the roots of tobacco seedlings. MeJA at the indicated concentrations was used to treat 4-week-old seedlings for indicated time, and the nicotine content was measured. Data are the mean ± *SD* from triplicate experiments. Bars with different letters are significantly difference at *p* < 0.05 (Tukey's test).

HT treatment induced a gradual increase in both JA and JA-Ile in tobacco roots, and the levels of JA and JA-Ile gradually increased after HT treatment and remained high for at least 9 days of treatment (Figure 4A). We also found that the application of exogenous MeJA treatment increased the nicotine content in tobacco seedlings (Figure 4B). These data correlated with the above finding that HT increased the transcripts of genes associated with JA biosynthesis including *NtLOX, NtAOS, NtAOC*, and *NtOPR* (Figure 3C). Diethyldithiocarbamic acid (DIECA) JA biosynthesis in plants. Application of DIECA partly suppressed HT-induced nicotine biosynthesis (Supplementary Figure 3), which further supports the role of JA signaling in HT-induced nicotine biosynthesis.

### High temperature promotes NtJAZ1 degradation

**Figure 5:**
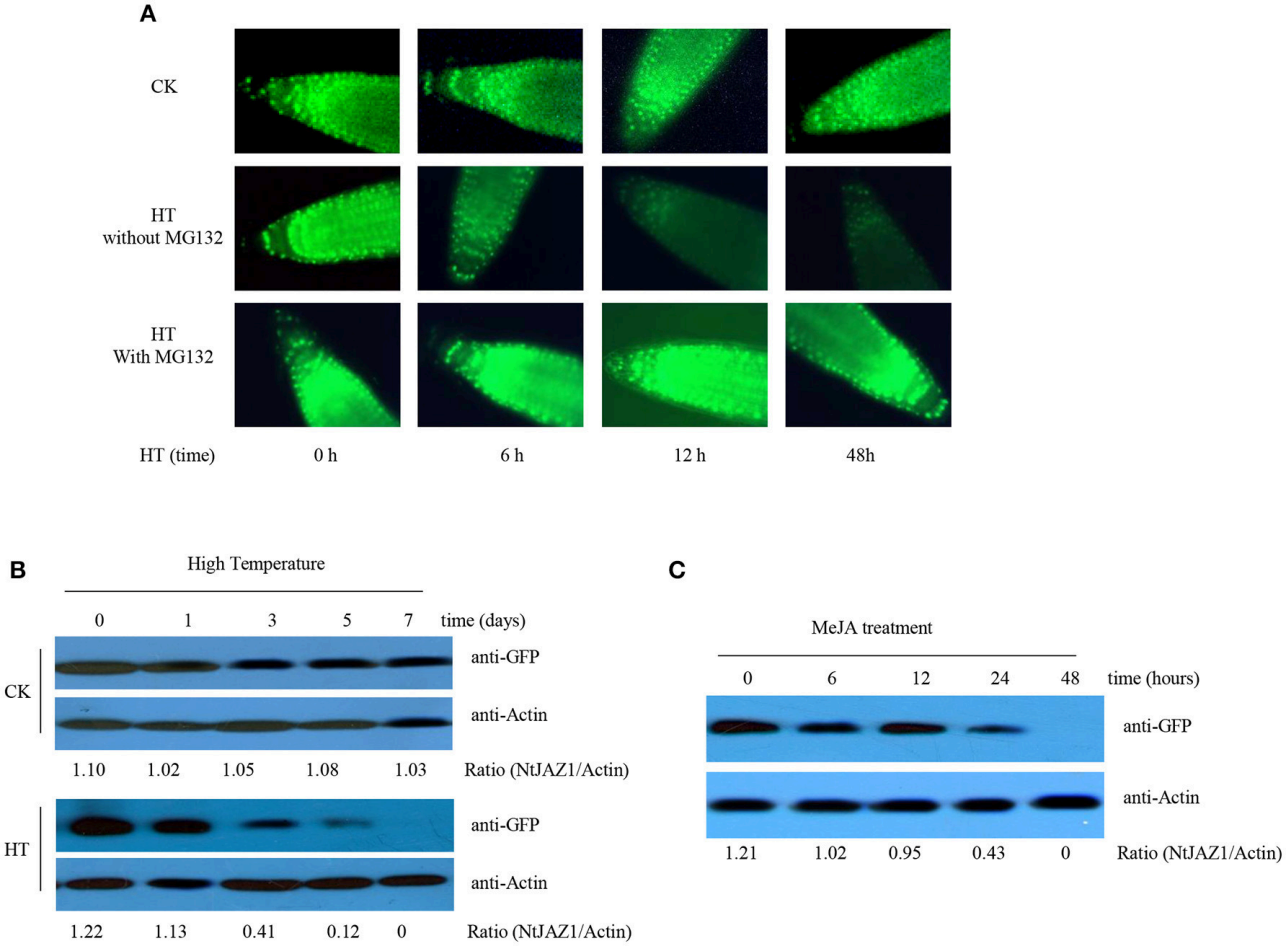
**HT and MeJA induce the degradation of NtJAZ1-GFP. (A,B)** HT induces NtJAZ1-GFP degradation. Four-week-old transgenic *NtJAZ1-GFP* #2 seedlings were subjected to HT treatment at 32°C or under normal condition at 24°C (CK) for the indicated times. The degradation of *NtJAZ1-GFP* was monitored by GFP fluorescence intensity. For proteasome inhibitor MG132 treatment, MG132 at 10 μM were used. **(B)** western blotting analysis of the stability of *NtJAZ1-GFP* after HT treatment at 32°C or under normal condition using anti-GFP. Anti-Actin was used as a loading control. **(C)** MeJA treatment induces the degradation of *NtJAZ1-GFP*. Two-week-old transgenic *NtJAZ1-GFP* #2 seedlings were exposed to 100 mM MeJA for the indicated times. Degradation of NtJAZ1-GFP was monitored by western blotting as in **(B)**.

JA promotes the degradation of NtJAZ1, which also participates in nicotine biosynthesis (Shoji et al., 2008). As HT induced JA accumulation, it is possible that HT also triggers the degradation of NtJAZ1 in tobacco roots. To test this hypothesis, we generated transgenic tobacco plants that constitutively express a GFP fusion of NtJAZ1 driven by the cauliflower mosaic virus (CaMV) 35S promoter (*NtJAZ1-GFP* lines). Western blotting using GFP antibody showed a higher level of *NtJAZ1-GFP* in the root tissue of transgenic *NtJAZ1-GFP* lines as compared with the non-transgenic control line (Supplementary Figure 4), confirming that a higher level of *NtJAZ1-GFP* is present in the transgenic *NtJAZ1-GFP* lines. Obvious GFP fluorescence was also detected in the root tips of transgenic *NtJAZ1-GFP* plants (Figure 5A). The degradation of NtJAZ1 was observed by monitoring the GFP signal in *NtJAZ1-GFP* lines. The intensity of GFP fluorescence in the roots of *NtJAZ1-GFP* plants decreased gradually during HT treatment (Figure 5A, middle panel) in contrast these lines under normal condition as the control (Figure 5A, upper panel). Consistent with this, immunoblotting analysis using anti-GFP demonstrated that HT obviously induced NtJAZ1-GFP degradation in the tobacco root tissue compared with those line under normal condition as the control (Figure 5B).

Proteasome-mediated ubiquitination modulates JAZ degradation in Arabidopsis (Chung et al., 2008; Yan et al., 2013). We thus investigated whether NtJAZ1 degradation under HT treatment also depends on the proteasome-mediated ubiquitination pathway. We treated a *NtJAZ1-GFP* line with MG132, a specific inhibitor of the 26S proteasome, to suppress the proteasome-mediated ubiquitination pathway, and found that HT did not efficiently reduce GFP fluorescence in the root tips of the MG132-treated *NtJAZ1-GFP* plants (Figure 5A, bottom panel), which suggests a role for the proteasome-mediated ubiquitination pathway in HT-induced NtJAZ1 protein degradation. Application of exogenous MeJA also accelerated NtJAZ1-GFP degradation in the tobacco root tissue (Figure 5C).

### HT enhances the binding of NtMYC2a to the promoter of NtPMT1

**Figure 6:**
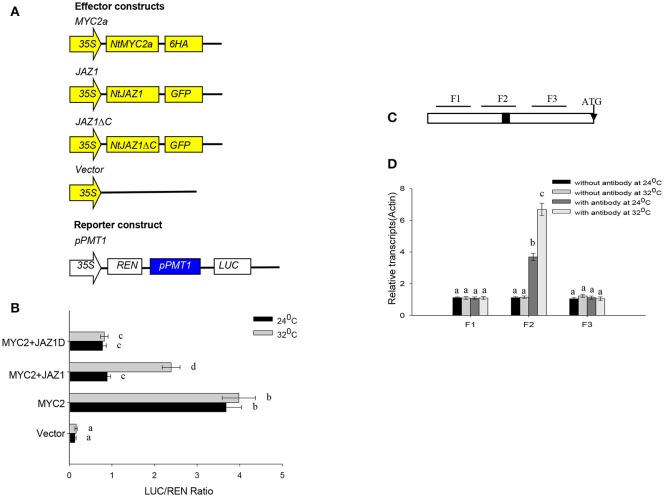
**NtMYC2a binds to the promoter of ***NtPMT1***. (A)** Schematic diagrams of the *pPMT1* reporter constructs and the *NtJAZ1-GFP, NtJAZ1*Δ*C-GFP* lacking the JAS domain.*pPMT1* and *NtMYC2a* empty effector constructs used in the protoplast transcriptional analysis system. **(B)** Expression assay for the *pNtPMT1:LUC* in tobacco protoplasts transformed with the *NtJAZ1-GFP, NtJAZ1*Δ*C-GFP* or *NtMYC2a* plasmid or empty vector. The luciferase/REN ratio is shown. Data are the mean ± *SD* from triplicate experiments. **(C)** Promoter structure of *NtPMT1* gene and the fragment used in the CHIP assay. The black box indicates the G-box motif. Lines indicate the regions (F1, F2, and F3) selected for PCR analysis following ChIP, the detail sequences information and primers sequences for amplifying F1, F2, and F3 fragments show in Supplementary File 2 and Supplementary Table 1. **(D)** ChIP enrichment showing the ability of MYC2a transcriptional factor to bind *in vivo* to the promoter region of *NtPMT1*. ChIP results were normalized to input chromatin, and a fragment in the *ACTIN2* promoter was used to normalize the data. Data are the mean ± *SD* from triplicate experiments. Bars with different letters are significantly difference at *p* < 0.05 (Tukey's test).

NtMYC2a can bind to the G-motif within the promoter of *NtPMT2* to activate *NtPMT2* expression (Shoji and Hashimoto, 2011). We also searched on G-box in the *NtPMT1* (Supplementary File 2). To further investigate the effect of HT on the binding capability of NtMYC2a to *NtPMT1 in vivo*, we generated a construct in which the luciferase gene is driven by the *NtPMT1* promoter and co-transformed this construct into tobacco root protoplasts along with the *NtMYC2a-6HA* construct by polyethylene glycol-mediated co-transformation. We observed significant luminescence in tobacco root protoplasts co-expressing *pNtPMT1:LUC* and *NtMYC2a-6HA* (Figures 6A,B); however, the luminescence intensity increased after the protoplasts were subjected to HT at 32°C for 30 min. Luminescence was observed in the two controls, root protoplasts co-transformed with *pNtPMT1:LUC* with the empty effector vector and protoplasts coexpressing *NtMYC2a-6HA* and *pNtPMT1-M1:LUC*, which harbors a mutation (CACGCA) in the G-box motif of NtPMT1 (Supplementary Figure 5).

We also performed ChIP assays using the transgenic *MYC2a-6HA* line and anti-GFP Notably, our assays showed that, among the G-box–containing (F2) and non-G-box–containing (F1 and F3) regions, only the G-box–containing region was greatly enriched by anti-GFP in the ChIP assays (Figures 6C,D, Supplementary File 2). These results demonstrate that NtMYC2a specifically associates with the G-box region in the promoter of *NtPMT1*.

### HT releases free NtMYC2a from the NtMYC2a-NtJAZ1 complex

**Figure 7:**
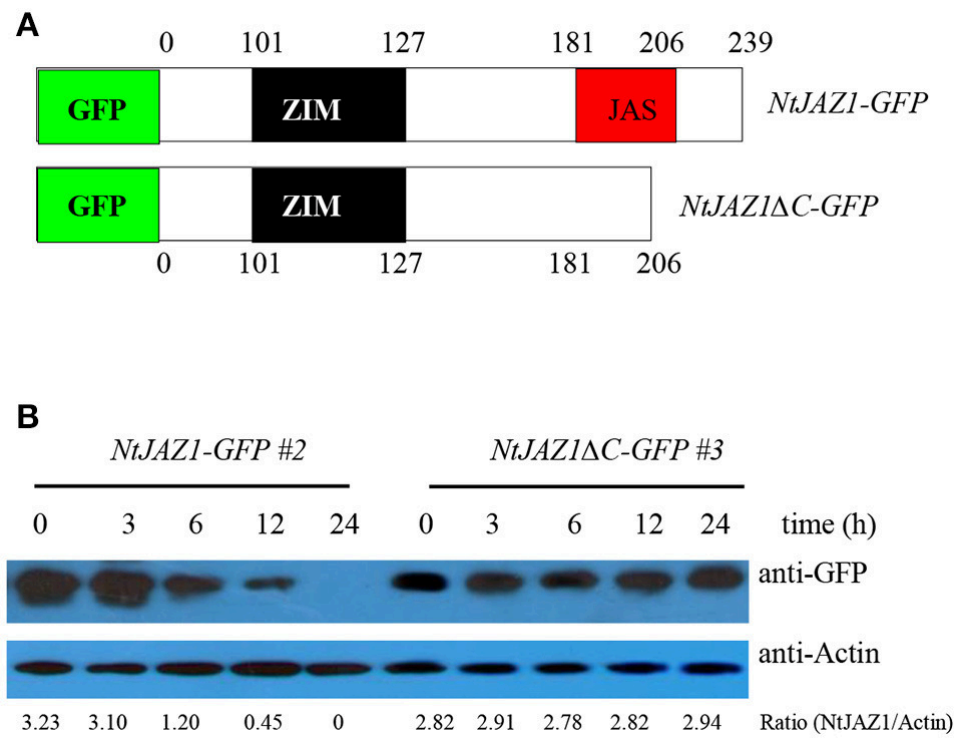
*****NtJAZ1*** and ***NtJAZ1***Δ***C*** suppress the ***NtMYC2a***-mediated up-regulation of ***NtPMT1*** transcription. (A)** Schematic diagrams of the fused *NtJAZ1-GFP* and *NtJAZ1*Δ*C-GFP* lacking the *JAS* domain. **(B)** Evaluation of *NtJAZ1-GFP* and *NtJAZ1*Δ*C-GFP* stability in transgenic *NtJAZ1-GFP* and *NtJAZ1*Δ*C-GFP* lines, respectively, under HT conditions. The transgenic *NtJAZ1-GFP* #2 and *NtJAZ1*Δ*C-GFP* #3 lines were treated with HT for the indicated times, and the stability of *NtJAZ1-GFP* and *NtJAZ1*Δ*C-GFP* was monitored using anti-GFP. Anti-Actin was used as the loading control.

NtJAZ1 can sequester NtMYC2a under normal conditions (Zhang et al., 2012). In addition, JA treatment can promote NtJAZ1 degradation to release free NtMYC2a and subsequently activate the transcription of NtPMT1 and nicotine synthesis (Shoji et al., 2008). To investigate whether HT treatment also releases NtMYC2a from the NtMYC2a-NtJAZ1 complex to activate nicotine synthesis, we generated a construct containing GFP fused to *NtJAZ1* (*NtJAZ1-GFP*) or to a version of *NtJAZ1* that lacks the JAS motif (*NtJAZ1*Δ*C-GFP*; Figure 7A). NtJAZ1ΔC is resistant to proteasome-mediated degradation (Shoji et al., 2008). We also generated a transgenic line expressing *NtJAZ1*Δ*C* driven by the 35S promoter (*NtJAZ1*Δ*C-GFP*). Western blotting indicated a high level of NtJAZ1ΔC-GFP accumulation in these transgenic lines (Supplementary Figure 4). HT treatment obviously induced the degradation of *NtJAZ1-GFP* in the root tissue of transgenic *NtJAZ1-GFP* lines but did not induce the degradation of *NtJAZ1*Δ*C-GFP* in the root tissue of transgenic *NtJAZ1*Δ*C-GFP* lines (Figures 7A,B). Consistence with it, we found that co-expression of NtJAZ1ΔC also repressed NtMYC2a-induced *pNtPMT1-LUC* activity at 24°C. In contrast, HT treatment abolished the inhibitory effect of NtJAZ1 on NtMYC2a-induced *pNtPMT1-LUC* activity but did not significantly inhibit the effect of NtJAZ1ΔC on NtMYC2a-induced *pNtPMT1-LUC* activity (Figures 6A,B), which suggest the possibility that HT accelerated the nicotine biosynthesis through releasing the inhibitory effect of NtJAZ1 on NtMYC2a function.

**Figure 8:**
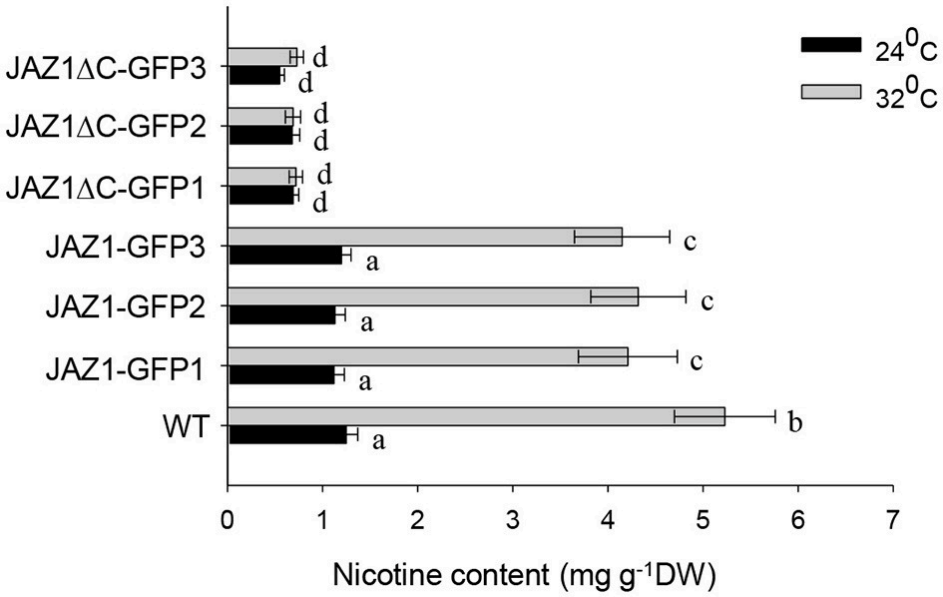
**Overexpression of ***NtJAZ1***Δ***C*** results in the suppression of HT-induced nicotine biosynthesis**. Three individual transgenic *NtJAZ1-GFP* and *NtJAZ1*Δ*C-GFP* plants were subjected to normal conditions or HT treatment, and the nicotine content in the roots was measured after 5 days of treatment. Data are the mean ± *SD* from triplicate experiments. Bars with different letters are significantly difference at *p* < 0.05 (Tukey's test).

The root tissue of *NtJAZ1-GFP* and *NtJAZ1*Δ*C-GFP* seedlings exhibited lower nicotine biosynthesis under normal conditions at 24°C compared with that of the non-transgenic tobacco. HT treatment at 32°C for 5 days obviously increased nicotine biosynthesis in the root tissue of the non-transgenic line. HT also increased the nicotine content in the *NtJAZ1-GFP* lines, in contrast, HT treatment did not significantly induce nicotine biosynthesis in the *NtJAZ1*Δ*C-GFP* lines as compared with that in the *NtJAZ1-GFP* or non-transgenic control lines (Figure 8). These data indicate that NtJAZ1ΔC, as the dominant-negative form of NtJAZ1, suppresses HT-induced nicotine biosynthesis after HT treatment.

## Discussion

JA signaling has multiple roles in the plant defense response against pathogen infection and insect attack and controls diverse developmental processes, such as stamen development, root growth, trichome formation, and secondary metabolism (Facchini, 2001; Farmer et al., 2003; Yang et al., 2012). Recently, JA was reported to function in the plant response to abiotic stress; for example, cold stress enhances Arabidopsis tolerance to freezing stress by activating JA signaling and inducing the ICE1-CBF pathway (Hu et al., 2013). In this study, we found that HT treatment obviously induced the accumulation of JA and JA-Ile in tobacco root and that this effect was more striking than the effect of other stresses, such as cold, salinity, and heavy metal stress. JA signaling is involved in nicotine biosynthesis in tobacco (Shoji et al., 2008). In our experiment, we also found that HT induced nicotine accumulation and up-regulated *NtPMT1* transcription and accumulation of its protein. It is possible that HT-induced JA biosynthesis triggers nicotine biosynthesis, as pretreatment with the JA biosynthesis inhibitor DIECA (Farmer et al., 1994; Hu et al., 2003) markedly suppressed HT-induced nicotine biosynthesis, whereas the addition of exogenous MeJA promoted PMT1 accumulation and nicotine biosynthesis. These observations support the notion that HT induces JA biosynthesis, which subsequently triggers nicotine biosynthesis. In agreement with this, a previous study showed that silencing of JA signal receptor *NtCOI1*, which blocks the JA signaling pathway in plants, suppresses JA-responsive nicotine biosynthesis in tobacco (Shoji et al., 2008). It is possible that JA signal transduction also play the role in HT-induced nicotine biosynthesis. Thus, our data demonstrate that *de novo* JA biosynthesis is necessary for nicotine biosynthesis.

NtMY2a belongs to the basic helix-loop-helix transcriptional factor. In tobacco, there are at least 23 bHLH subfamilies containing 190 bHLH genes were identified (Rushton et al., 2008). The transcription factors NtMYC2a and NtMYC2b form nuclear complex with the NtJAZ1 repressor to regulate nicotine biosynthesis (Zhang et al., 2012). Silencing of NtMYC2b in tobacco hairyroots strongly decreased transcript levels of jasmonate-responsive structural genes, including those involved in nicotine biosynthesis (Shoji and Hashimoto, 2011). Here we found that HT treatment increased the transcriptional level of NtMYC2a. The ChIP experiments further confirmed that NtMYC2a can recognize and specifically bind the G-motif within the promoter region of *NtLOX, NtAOS, NtAOC*, and *NtOPR*, all of which are the key genes associated with JA biosynthesis in tobacco. HT treatment also increased the transcriptional levels of *NtLOX, NtAOS, NtAOC*, and *NtOPR*, which agrees with the finding that HT induced JA and JA-Ile accumulation. It is possible that HT treatment increased the transcriptional levels of NtMYC2a, which then bound to the G-motif of JA biosynthesis–associated genes including *NtAOS, NtAOC*, and *NtOPR* to activate their expression, ultimately resulting in JA and JA-Ile accumulation.

In Arabidopsis, JAZs function as important transcriptional repressors in the JA signaling response (Chini et al., 2007). The JAZ proteins contain the ZIM motif and the highly conserved JAS motif, and most JAZs are rapidly degraded by MeJA application, insect herbivory, and mechanical wounding in a COI1-dependent and 26S proteasome-dependent manner (Steppuhn et al., 2004; Chung et al., 2008). In tobacco, 12 members of JAZ family have been identified in tobacco, these full-length NtJAZs proteins could be clustered into five main subgroups by phylogenetic analysis (Oh et al., 2012). JA signaling induces degradation of the NtJAZ1 proteins, which stimulates nicotine biosynthesis, and this effect is abolished in transgenic COI1-silenced tobacco plants (Shoji et al., 2008). Here, we found first that HT treatment accelerated the disappearance of GFP fluorescence and NtJAZ1-GFP degradation in the transgenic *NtJAZ1-GFP* line, an effect that could be blocked by MG132. Second, the truncated form of NtJAZ1ΔC, which lacks the JAS domain, showed resistance to HT-induced protein degradation in the transgenic *NtJAZ1*Δ*C-GFP* lines. These data indicate that NtJAZs are subjected to 26S proteasome-mediated degradation under HT treatment. AtJAZ proteins can bind to and inactivate a set of transcription factors, such as *AtWRKYs, AtMYCs*, and *AtICE1*, and the JA signal can activate these transcriptional factors (Hu et al., 2003; Zhang et al., 2012; Qi et al., 2014). Tobacco JAZ proteins can be rapidly degraded after MeJA treatment, whereas the truncated form lacking JAS motif did not. Transgenic plants expressing the non-degradable JAZ form did not efficiently accumulate nicotine after MeJA treatment. Consistent with this, we found that HT induced the degradation of NtJAZ1-GFP in the transgenic *NtJAZ1-GFP* lines but did not induce the degradation of NtJAZ1ΔC-GFP in the transgenic *NtJAZ1*Δ*C-GFP* lines. Our *in vivo* luciferase activity analysis demonstrated that NtJAZ1 suppressed NtMYC2a-induced NtPMT1 transcription and that this effect was reversed by HT treatment. Additionally, the transgenic expression of *NtJAZ1*Δ*C* suppressed *NtPMT1* transcription, and this effect was not reversed by HT treatment. As HT led to the efficient degradation of NtJAZ1, but not of NtJAZ1ΔC, which lacks the JAS domain and is resistant to COI1-dependent degradation, HT may disrupt the interaction between NtJAZ1 and NtMYC2a to activate the expression of *NtPMT1* and thereby nicotine biosynthesis. In contrast, HT cannot disrupt NtMYC2a and NtJAZ1ΔC binding, thereby maintaining NtMYC2 in an inactive state. In agreement with this, we found that the transgenic *NtJAZ1-GFP* and *NtJAZ1*Δ*C-GFP* lines produced lower levels of nicotine than the wild type under normal conditions, but that only the *NtJAZ1*Δ*C-GFP* line continued to exhibit reduced nicotine biosynthesis under HT treatment. Similar to the non-transgenic line, HT treatment also up-regulated NtPMT1 transcription and nicotine biosynthesis in the *NtJAZ1-GFP* line, as HT efficiently degraded NtJAZ1 and thereby removed its inhibitory effect on NtMYC2a-induced *NtPMT1* transcription. In agreement with our results, a previous report showed that transgenic *NtCOI1-RNAi, NtJAZ1*Δ*C-GUS*, and *NtJAZ3*Δ*C-GUS* lines exhibited lower levels of NtPMT1 transcripts and of nicotine (Shoji et al., 2008).

**Figure 9:**
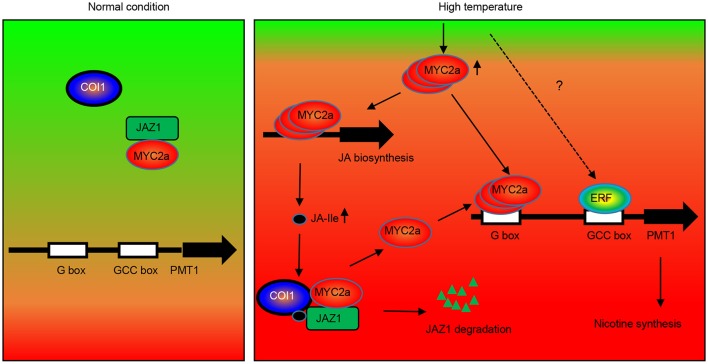
**Hypothetical model of the function of NtJAZ1 and NtMYC2a in HT-induced nicotine biosynthesis**. Under normal conditions, NtMYC2a forms a complex with the NtJAZ1 repressor and NtMYC2a cannot bind to the promoter of NtPMT1 to activate its expression. HT conditions induce JA biosynthesis to generate JA and JA-Ile. The presence of JA-Ile promotes the interaction between JAZ1 and COI1 to induce JAZ**1** degradation via the 26S proteasome. As a result, free NtMYC2a is released to activate NtPMT1 expression by binding to the G-box element in the promoter region of *NtPMT1* and induce nicotine biosynthesis. Meanwhile, high temperature also increased the level of MYC2a transcripts, which bind the promoter regions of genes associated with JA biosynthesis to activate JA biosynthesis. Other transcriptional factors, such as ERF, may be involved in this process to synergistically activate NtPMT1 transcription with NtMYC2c.

To explain the molecular mechanism underlying HT-induced nicotine biosynthesis in tobacco, we propose the following simplified model (Figure 9). Under normal conditions, the NtJAZ1 repressors physically interact with NtMYC2a transcription factors, attenuating their transcriptional function and thereby repressing expression of downstream structural genes involved in nicotine biosynthesis. However, HT stress induces the production of endogenous JA-Ile, which is perceived by NtCOI1 as the key regulator in the jasmonate signaling pathway. NtCOI1 then recruits NtJAZ1 and mediates its degradation via the 26S proteasome pathway. As a result, NtMYC2a is released and activates the expression of *NtPMT1* and subsequently induces nicotine biosynthesis. Certainly, we cannot exclude other possible mechanisms in addition to COI1-dependent JAZ degradation, as the ability of JAZs to bind MYCs is also modulated by other factors, such as DELLAs (Qi et al., 2014). Furthermore, chromatin remodeling factor HAD6 and ethylene-responsive factor ERF19 also influence the level of MYC2a-induced *NtPMT1* transcription (Shoji and Hashimoto, 2011). Taken together, our data reveal the novel molecular mechanism underlying nicotine biosynthesis in tobacco plants subjected to HT.

## Author contributions

LMY, JL, and XH designed the research; LMY, JL, and JJ performed the research; LMY, PL, LLY, YL, EA, LH, and XH analyzed the data; LH and XH wrote the article.

### Conflict of interest statement

The authors declare that the research was conducted in the absence of any commercial or financial relationships that could be construed as a potential conflict of interest.

## Supplementary material

Supplementary Figure 1 **Quantitative RT-PCR and western blotting analysis of the transgenic lines overexpressing ***NtMYC2a-GFP***. (A,B)** Three individual transgenic lines overexpressing *NtMYC2a-GFP* and a non-transgenic wild-type line were analyzed by quantitative RT-PCR **(A)** and western blotting **(B)**. Anti-ACTIN was used as the loading control.

Supplementary Figure 2 **Differential transcriptional level of JA biosynthesis related gene in NtJAZ1-GFP and wild type line**.

Supplementary Figure 3 **Effect of the JA biosynthesis inhibitor DIECA on HT-induced nicotine biosynthesis**. Two-week-old seedlings were treated with HT (32°C) or with HT plus 0.5 mM DIECA for the indicated periods, and the nicotine content was measured.

Supplementary Figure 4 **Western blotting confirms the expression of NtJAZ1-GFP in the transgenic ***NtJAZ1-GFP*** lines (Left panel) and NtJAZ1ΔC-GFP in the transgenic ***NtJAZ1***Δ***C-GFP*** lines (Right panel)**.

Supplementary Figure 5 **NtMYC2a binds to the promoter of ***NtPMT1***. (A)** Schematic diagrams of the *pPMT1 and pPMT-M1* as the reporter constructs, the *NtMYC2a-GFP* and the empty vector as the effector constructs used in the protoplast transcriptional analysis system. As for the *pPMT1* reporter vector, the blue box in the *NtPMT1* promoter means the G-box containing CACGTT, while the black box in the *NtPMT1* promoter means the mutated G-box containing CACGCA. **(B)** Expression assay for the *pNtPMT1:LUC* or *pNtPMT1-M1:LUC* in tobacco protoplasts transformed with the *NtMYC2a-GFP* plasmid or empty vector. The luciferase/REN ratio is shown. Data are the mean ± *SD* from triplicate experiments.

Supplementary File 1 **The sequences information for the promoter of ***NtLOX, NtAOS, NtAOC***, and ***NtOPR***, respectively**. Different G-box in the promoter are highlighted by different color.

Supplementary File 2 **The sequence information for the promoter of ***NtPMT1*****. The G-box motif were highlighted, and the F1, F2, and F3 fragment for CHIP assay were labeled by different color.

Supplementary Table 1 **Primers used in this study**.

## Abbreviations

AOC: allene oxide cyclase
AOS: allene oxide synthase
COI1: CORONATINE INSENSITIVE1
HT: high temperature
JA: jasmonic acid
LOX: lipoxygenase
NtPMT1: N-methyltransferase 1
OPR: 12-oxophytodienodate reductase
JAZ: JASMONATE ZIM DOMAIN.
