# Supplementary material for: High Temperature Induces Expression of Tobacco Transcription Factor NtMYC2a to Regulate Nicotine and JA Biosynthesis
Source: Front Physiol. 2016 Oct 27;7:465. doi: 10.3389/fphys.2016.00465 (PMC5081390; doi:10.3389/fphys.2016.00465)

Supplement Figure 1

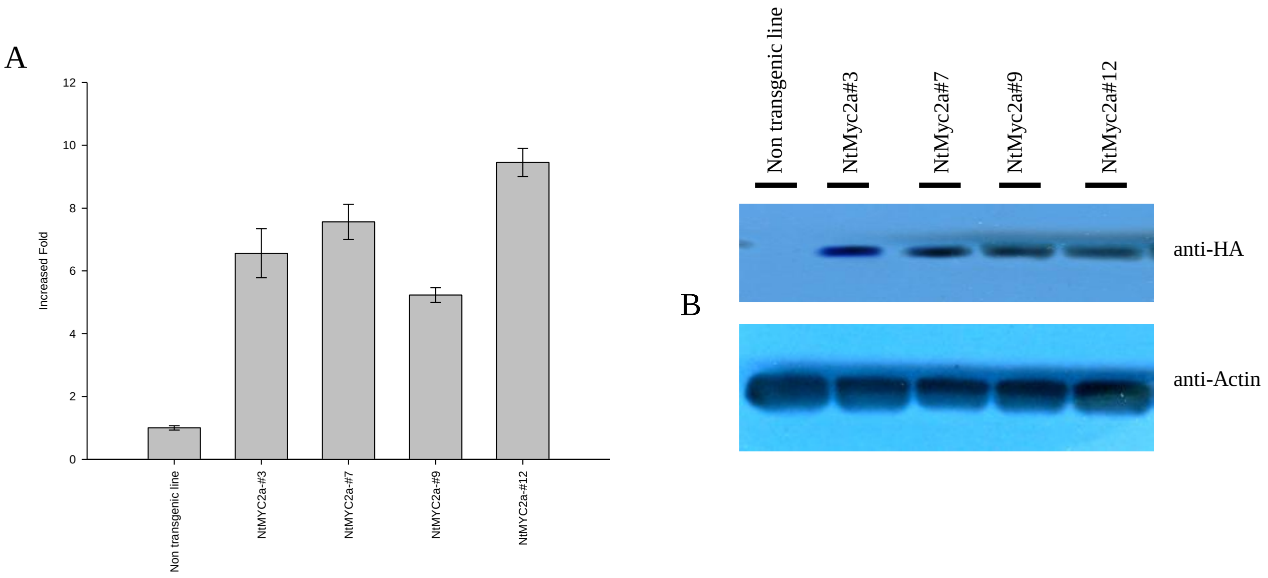

Supplement Figure 2

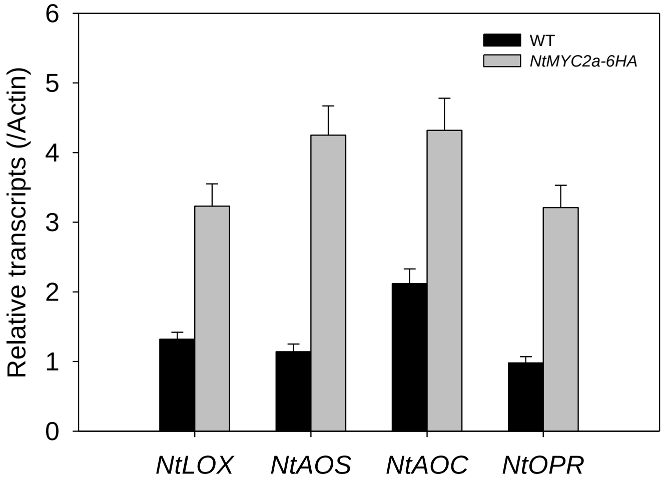

Supplemental Figure 3

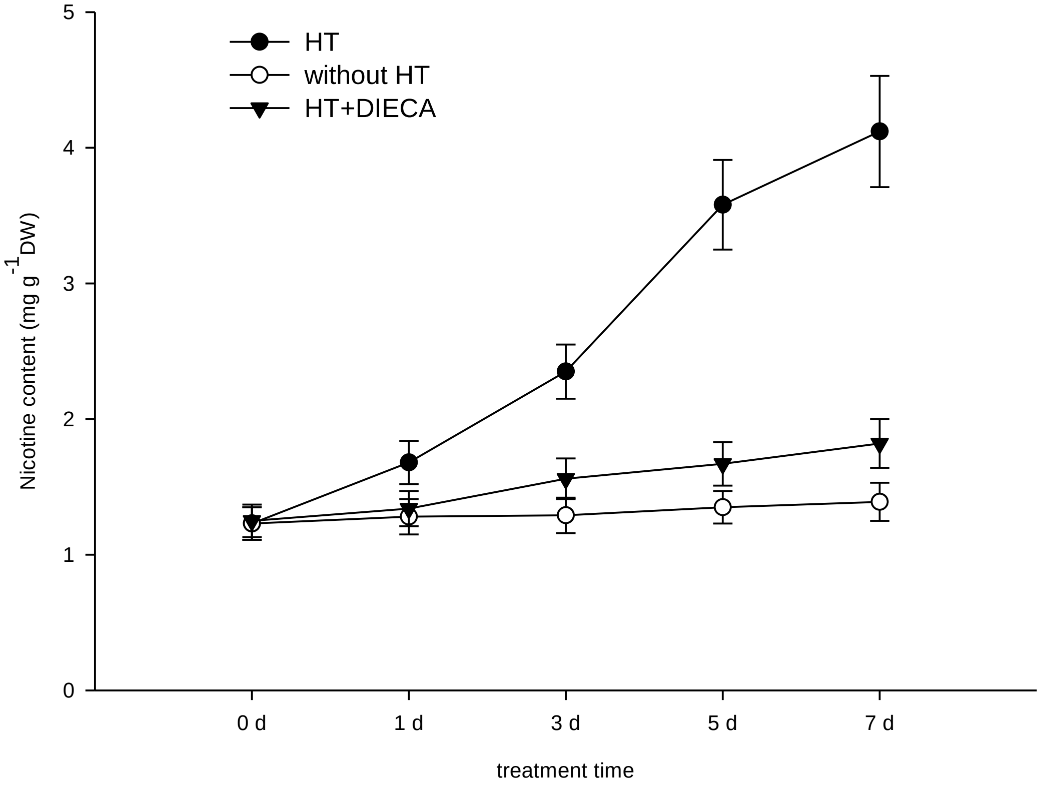

Supplemental Figure 4

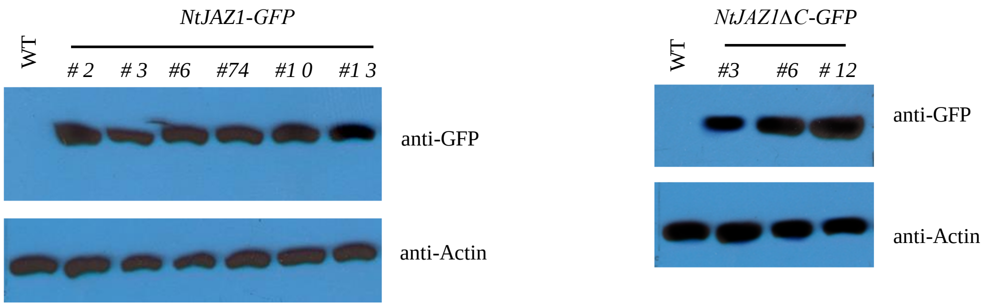

Supplemental Figure 5

A

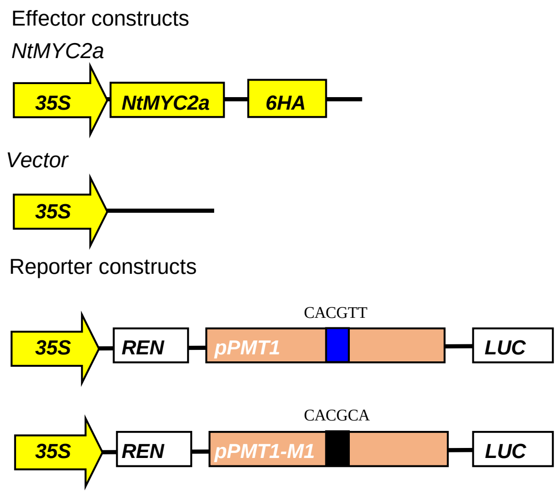

B

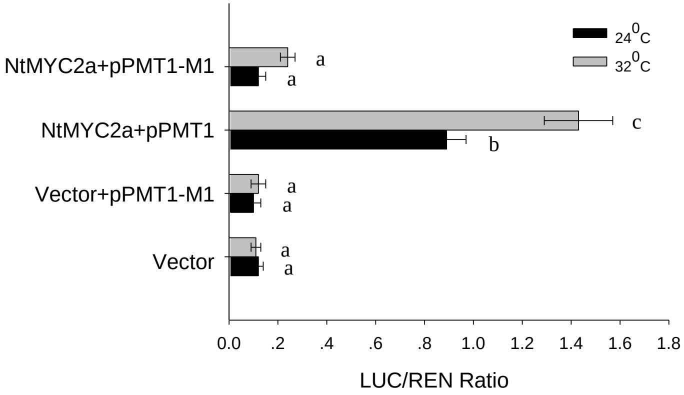

Supplement: Supplementary Figure 1 — Quantitative RT-PCR and western blotting analysis of the transgenic lines overexpressing NtMYC2a-GFP. (A,B) Three individual transgenic lines overexpressing NtMYC2a-GFP and a non-transgenic wild-type line were analyzed by quantitative RT-PCR (A) and western blotting (B). Anti-ACTIN was used as the loading control. [file Presentation1.PDF]
