# Supplementary material for: High Temperature Induces Expression of Tobacco Transcription Factor NtMYC2a to Regulate Nicotine and JA Biosynthesis
Source: Front Physiol. 2016 Oct 27;7:465. doi: 10.3389/fphys.2016.00465 (PMC5081390; doi:10.3389/fphys.2016.00465)
Supplement: Supplementary File 1 — The sequences information for the promoter of NtLOX, NtAOS, NtAOC, and NtOPR, respectively. Different G-box in the promoter are highlighted by different color. [file Presentation2.pdf]

>Ntab0305800 Nicotiana tabacum 3KB Upstream sequence, expressed protein, similar to Probable linoleate 9S-lipoxygenase 5 OS=Solanum tuberosum GN=LOX1.5 PE=2 SV=1
CAAAACTTGACAAGTAACTACAGCAGTATTAAATGTTGTAGCCTTTTAGGTGCCAACAAT
AAATATTTTGGGGAAGAGGCTGTTTGTGATCATACTCATATAACATAAACTTAGCCGCGT
GCATTATATATGACCGTCCTCGTCTTGCCTATTAAATTAGAGTATGATCTTCAATCTCTC
TAAACATCACCTATCACTATATATTGAGGCTATCCATTGTCATTTACCTCTATTTGACAA
TTTCATACTATAATATAATTACTCATGAATAGTTTGTATATTCAGACAAATTTTGAGTTC
TCTAAAGCTGGGGATTCTTTAATCTCACATGTACATACATTGACCTACAAACTTCTAACA
CAAAAAACGAATCTAATGTAAGTCTATTAACAACGACTAAGTTAATTTCTCCTTATAAGT
TTAGTGTCGCTTGTATAGATAGTTTAATTTGCTTGTGTTGTGTTATGTTCTTTGCTAGCT
AAATCTTCATGGATTCTAAAAGATTATAAGCCCTTTTTCTAGATTAATTAATTGCAATTT
TACTTCATGTGATTTTAAGTCGACTTCTTTGCTTTTAATGCCTTCAAATTATTATCGCGG
CGCATCTTATACAGATGCATTGTATTTGGATGTCTATAGAAGATGTAGCCTAACCATCTT
GGACCACATTCTCTCACTTTATCTTTTATGTACTACATAGAGGACAAGATATATACTTTT
CTAAAATTTTATCGTATAATCCGTAAATTTAATACCACGATACTTTACAGAATTAAGAAT
ATCGCCTTTGTTCTTATAAATCAGAGTAGAGGAGCTCTTTCTCCACTCGTTGGCATATTT
CATCTCCTTAATATAATATTAACTAATAATCTCGGTGAGCCATACTCTCTCAATCTCTCC
AAGTTGCTTTTAGACCTCTATAAGGATACTATTTGGAGTAAAACAAATTGCTAAACCCTC
ATATTTTTTTCCCTTTCATGACCATTTATTTCCCTATATATAGTTCTATGAGTGCATGTA
ACGCTCCCGTGTTGCACAAATATAAACGTATAAAATTATTCTCATAATTGAAAGTACATG
TATTTCTACAAAATAGAATTGTTTCTAGCTATAGTAAAATTCTCCATAGGAGAGTAACTA
AATTTAATCAAAATTTAGTCCATAGTTACGTTGGGTAGTGAAACTAGAATGGAGTGAACC
ATTACTAAGATTGTTATTCTTAAAAATGTCACTAGCAAATAGCAATAGGCTCTATATTCA
TATCAATAGCACGACACGCATCTTGTGATGAAAATTGTTGATTATTCAATCAAAATTTAA
TGCTCAACTATACTTATATGGAGATCCATCATTGACATCAATTGAGTTTTATATATAATT
TTGTACCATAGGTTGTATATATAAAATAATAAAATAGTATTGATGCATTTGAATAATAAA
AAAGAGAAGAGAAAGGATTTTGACAAGAAAGGCAAAAAAATTTCATAGAGGAATTGAACA
GAAAAATTAAAATATTAGGATTCAAATCACTCCACTTGTTTGCTTGTGGGGAAGAATTGA
CCACATAGACAACAAGAGAAAATAAATTTGAAATGCATTTAATTGGCTATGATCAGCTTC
CTTACTCATATCCTACAAGAAAAGAGGAAAAGTATTATTGGAAGGGGCTTTTCTTAGTTT
GAAAGTTCAGAAGAAGATGGCAAATGACTTTTCCAAGTCAGAGTCGAACATATGACTATT
TCCTAAAGCATTTTAATTTTTAATAAACACATATACAGTCAAACCTCTCTATAACATGTT
TGTTCCGAAATTTTTTGACTGCTATAGTAAAGTGTTGTTATAGAAAACATATATATTATA
ACATGCATAATATAATACTCGGTCCTGAGAAAAATTCGGATTTTAAAATAAATGACTATT
ATATAGGGATATTGTTATAGAGCAATCTGATTGTAGTAGCGTCGTACACTTAATCTAAAC
GATGCATGTGGGTAAATTTTGATGATAGACAAACTCAACGTCAAAAAAGTAATTAGAAAG
GCAATACACGAGTCTTATTAGGGTAAAAAAACATACTATAACTATTGAGGTAATCATCTG
ATTGAAATTACTATTATAAATTGTAACAATAGAACAACTATACGTAACCAAAGTGTTTTT
CTAAGAATGAGGGTTAATTACACGCACGCGGTGTTTATATCAAACAAATGAAGAAGAGCA
ATGGATAAATGTTCAAAAATGATAATTTTATTTACCATCTACATACAATGTGTAAATACT
TTCACATCACTCCTTGCAAGTTGCAACTAATTAACTAGCACAATTTTGTAATATTACAAC
AATTCTAATCGTTTTGAAATGAGATGCAAACGCATCAAATTACCTCGGCAAAGATGGAAT
TTCACGGCACAATATAAACAAAGCAAAACTACGGAGTTTGGACGATTTTGAGCGAATCGT
TTTTTTTTTGGGGGAAAAAAATATTTTTGAGGAAAAGCTTGTTTATGTTTTACGGATGTTC

CAAATATTAAACAACTTATTTTAGTGTAGAAATTAGATAAACATGAATAACAGATAATGA
ATAAGTATTTTCAGAATCACTCCAACTAAGATTGACGTGTAGATTTTTAGAAAGGTAAAA
GAAATTCATTTGGGCCCAAGAATTATCCACGTAAATAAACCTGTGCAGACATTAATTGCT
GTAAAATTTTATCCGCATGAGTATTTGTTTTAATAAATAAAAGGATAATAAAGACCAATA
TGGACAGACGATAGCTAGACAGGAAAGTTGGAATATAGAGATGGCTTTTGTATTTTTCTT
TTTTTCCCTTTTTTATTGTTTTTCTTTATTCTCAACTAGACAACTACTTGTTCTTGCTAT
AAATATCAGATTTCCTAGTTAGAAACTTCACAACACAAATTAAATTAAACTCATCTAAAT
TCTATTTTCTTTCGTTTTCTTGGAAGGTTATTGAGAGAGAAAGTTATCAAACAGTTAATA

>Ntab0018690 Nicotiana tabacum 3KB Upstream sequence, expressed protein, similar to Allene oxide synthase, chloroplastic OS=Arabidopsis thaliana GN=CYP74A PE=1 SV=3
TTATAGCCCCGGGCGAACACTGCGATACCAGCGTCATCATCCATGGAACGGTGGGCACGA
AGGGCGGTGGGTGGCTCTGATACCATTGATACAACCTAAGGAGAACCACACCCCAAAAGC
TAACTATTAAGGTGGGAGAGCCCAAGGCTATATAAACCCCACATCAGCACATTGCAATAC
GGATGTGGGACTCAAGTCTCATACCTTGCAATGGACCATAACACCTTATAGACTAAAAAA
GAAGATTATCTTATAGATTTTCTATCATCAAGATTCTCAATTTTTGTATTTTTAGCTTTG
CATGTTATTGTTGTTACTATTTATTTATTTTTGGATTCAGTCAATTTTAGGATCTCGTAT
GAGATGTACATAGATTATTTCTTTCGTTTCTTGCGGAGAGGATTCGGCCTTTGGACGTGA
CAAGAGAAAGAAGAGACGGGCTAGAGAAACAGAAAAACGAAGAAATACAGATTTTTTTTA
AAAAAAACTTGACCTAGGTGCATAATAAAATTGAATTTTAAGGTATATGCATAAAATTGA
ACACTGACACTGAACTTTGGAATCTCAAATTCAAGAAAGAAGTACCATATTGTACATACA
GTGATACATTTGTTAAAATGGTTCGTAAACAATTAAGCTCTTGGTAGTGATAATTTATTA
CTACTAATTTTGTTTCAATTTAGACGGCGCACTTTTCTTATTTGTCTGTTCTAAAAAAAA
AATATATTTCTACAATTAAAAATAATTAGAACTTTAAAATCTTAATTTTGGGACACCCCA
ATTTCCGATAAGGTGAATCGAGCAAGAGACGTGATGATAAGGAGCCGGAATCGAGGCAAG
TACCTTTTATAACCACACAAATGTCATGATTCCACAAAACTTTTAAGACAACAAATTTCA
AAAGTCTTTTTTTTCCTTAAAATCATGCCGAGCCAAACTATCTTATCTAAATTGAACTAC
TTTCTTATATATTTTGCCATGTGATACACAACCCCTCACAGAGGAGATTATCTTATGGAC
ATATCTAAAAGGAGATTATCTTATAGATTCTCTACTATCTTGCAAAGTTTGGATTGCCAA
CTTTGCACGTTGTTGTCGTCAGTTTTTTTTTTTAATTTTTAAATTCATATTAATTTATAGA
TTTTAGCATTTCATCTAAGATTTACATAAGATTTCATTTCTTGAGGATATGGTTCGATTC
TGACCTTGGATATGAAAGGAGAAGAGAAAGAAGAAACAAGCGTACCATAAAAAGAGAAGA
AATAAGAAATACTTTTGGTAAGATCAGAACAGCGTGACTAGTAAAATTAAACTTTTAACT
ATTAAGCTGTAATTTTCATCCTAATTTTAATTTCACCCCTTAAAAGTATAATATTTTCAA
AGAAAATTTTGAACTTTTTATTAACAGCTTATTTGGATGGTTGTTACGTATCGGTATTGT
TTTGATATATACAATATTTGAATAGATTATATTGTTCCCAATCGTTTCATGATGTCATGA
ACGAATAATATGAAGAATAAACTTGCAATATTATAAAGAAAATATAAGGTAAGGTACGAG
ATAAAATTATTTTATAAAAAAATAGGGTAAAGGATAAAATAAAATTATTTAATAATAACG
AAGGACAAGATGAGAGGAAAATATAAGGTAACGACGCGACCACACCAAATCGATCGTTCC
ATAAAATGATAATTTAACAATACAATACAATAAAATTTAAATTTCAATCAAAATAAATAT
TATCTTTAATCGTAACAATACAATAAAATACTACGTGTAACAACAATCCAAACAAGCAAA
TCAAACACCTACACACAAAACATATAATTAGACCTTGTTAAAATGATTGGAAAAATAAAA
TCCTACTCCTAGGAATTAAGACTTCTCGATTTATTATATTTTGCCATATGGTACACAAGT
CTGGCTCACAATTTTCAACGTTATATATCAGGGAATATTCAACGCCGACACTAGTCTAAC

ATTCAACTTCAACAACAGTTTCTTTACACCAACTTTTTAGTATATTACACGCGTGAAATT
TTATATACACACTCAAACACAAAAACACAAACATCAAACTTTTTCCAGTCTTACCACTAC
ATAAAAAGAAAGAAAATTTCATCAAATATCATCACACTCCCAGTCTCCCACTGCTTAAAG
TTCCACTTTCTTTTTTATCAAAAAATAATAATAATAATCATACTCCATTTTCTTACAACA

>Ntab0920680 Nicotiana tabacum 3KB Upstream sequence, expressed protein, similar to Allene oxide cyclase 3, chloroplastic OS=Arabidopsis thaliana GN=AOC3 PE=2 SV=1 TAAAGATGAAA
TGGTGAAAAAAGGTCAACGTTATAAATGATTCAATGATATACTATTGCAAACTTTAACAT
ACTATTACTAATTACCATAGTATTTATTTATATTAAGCAAACATTATTATACAGACATAA
GAAATAGTTTGATTTTTTTTAATTGTATTGCATAGCAATTAATGAAAGATTTTAATAAAG
TTTCATATTAAATAAAATTTCTGTGAATAGCAATAAAGTATTAAATATACTTTTAGTATTTCTTATACATATGAATC
ATTATTGAAATTTCAATTAGTATAAAAAATGATTTGAGCTAATGTATTTTTATTATGTTTAATATTATTAGTTCATT
TGAAAAAAATCCCTTAATAATGAAATATTGTTTAACGTAAGTCATGTATGTATATAACTAATGATA
ACAAAAGTAATAAATGCATGTCATATAGCAAGTACCATGTAAAATGAAAAAAATATTTAT
TTTAAAAATAAAAAAGAAATAAATACTAACATTGATGAAAAAAATAATATTATACATGTC
ATATAGCAAGTACCATGTAAAATGAAAAAAATATTTATTTAAAAAAAAAAAAAAATACTAA
CATTGATGAAAAAAATATATTATACATGAAATAAAAACATCATAAAGATGCTAGTTATTA
AACTAGTCAGTTACGCCTAGAAGAATTATTGGTGGGCCCACATAATATATATAGAGTCTT
TCAAAAAGGCTTTATACTTATAGCGCATTTCAAAAAGGTTTTATACATATAGCGCATTTT
AAAGGGACGCTATACTTAACTGGGCAAACGACCATTAAGATATAACGCCGTTTAAAAGAG
CGTTATATATAAAATGGTCACCCAATTTTTTTCACACATTTTGGTTATTTGAGTCTAAAA
GAACCATATTTTGGTTCCGGCCTCCTAGAACTCACAACTTTAGGACATGAAGACTAATAG
CCTGTTTAGCCAAGTTTTTTTTGGTCAAAAGTTTTTTTTTCTTTTCCAAAAGTGCTTTTG
GCCAAAAAATTAAGGTGTTTGGAAAAGTTATTTTGTGGAGGAGCAGAAGTAGTTTTTGAGA
AAAAGAAAAAATATAGTTTCTATTCAAAAGTATTTTTGTGAGAAACACTTTTGAGAAAAAT
ACACTCAGAAGCAATTTTCAAAAATTTGATCAAATACTAATTATGTTCAAAAGTATTTTT
TAAATTAATTAATCAAACACAAATTGATTCTTATTAAAATTATTTTTTTGAAAGTCATTT
TTAAAAAAAAAAAACACTTATAAAAAAAAAGCAGATTTTAGAAGTTTTGACGGATTACAAG
TCAAAGTCACAAAGCGAGTTTCCCCGTAGGCCAATCCAAAGCGGCACAGACCACCAAGAA
CAAAAAACCACGTGGAGACACTATTTCCCACATTGGCAAAACATTGTTTGTTTATTCCTA
GAGAAGACACCTCGAAAGCGTCTTTGACTATTCCCAATCTTCCAGTCTCCACTCTCCACA
CACACAAAACTCCCATTAACACATCGTACACCGTCCATCAAATCATGAATCACAGATCTT
ATACTAATAATAATTTTATGATATTCCCATTTTCCACACATGGAATTTGGACCCCTCAGC
ACTATATAGTTAATCACCTTTCATTTCATCAGCCTCAACAACCCCATTCAACTTTCTATT
CTAGTCATTTATTTTCCCCTAAAAAAACCAACACATTTGCAAGTTCATTCCTCAGCAGCT

>Ntab0512300 Nicotiana tabacum 3KB Upstream sequence, expressed protein, similar to 12-oxophytodienoate reductase 3 OS=Solanum lycopersicum GN=OPR3 PE=1 SV=1
NNNNCCCCTCCCCACCCTACCCCACCCCACCCGAGAACATGACCTTATTCCACCATCACT
TACCCCAGCCATTATAATCTACAGAAAATTACGCTAGAACTACCGCTAAAACAGACAAAG
GATTAAACTAAAGGAGGCAGGTCTAACACTTACAACTCTAACCACTAGAAATGCGTACAA
ATAAGACAAATAGATAATCAAGAATTAAACAAGTGATAACAATATCTCTAATAAAAGGCC
AACAGAACAGCTACTAAGAAATCAAATTACAGTCTAGAAATCCCAAAATAGTTATTAGCA
AGAAAAATATCTAGGCAGATGCACTAAATAATTATAGGCAATCAAGAATTAGGCTTAGTG

GGCGTTTGGACATAAGATTTGTAAAATTCAAAAATAGAGGGAAAAAATTTCAAGTGAAAA
TGGTATTTGAAATTTAAAGTTGTGTTTGGAAATGATTATAATTTTAGGTTTTTTTGAAGT
TTTGTGAGTGATTTGAGTGAAAATTTTGAAAAACAACTTTTTGGAGTTTTTCAAATTTTC
GAAAATTTCCAAAATGTATCTTCAAGTGAAAATTGAAAATTTTATGAACAAACGCTGATT
TTGAAAAAAAAGTGTTTCTTTTTTGAAAAAAAAAAAGAAAAATTTCTTATGTCCAAAAGG
GCTCTTAAACTAACGCAAACTAATTGGATACTAACAAGGGAGACAAAGATATTAAACGAA
AAACAGAAGGTACCAACTCCTGATTAGTGCGAAACCTAGGTAAATCGTGGTAAATGTCAA
GGTCATGCTACCGCATAACCTTAGTCAATTACTAATATATTACTATTTTCTAAAAATATA
TATATTTGTATATATAAAAAGTTTATCGAATTTTATGGATGCTTTTGACCCCTTTACCTA
TAGCATCGGTCCGCGTCTGTGGTCAAATATAATAAAACTTTCTCGGATAAGTGAAGTGAA
TTTTTTTTAATAAAAATTGCGGTAAGATTCGCCGCGAACAAAGACAAGACCTATGCATAC
AAATGAATTTATTCCACCGTACACAAGTCACAAGTGTCCGTCAATAATGTAGACAAACGT
GCAAATTTTAGTATAATAGAATTTAACAATAATAAAAATAAAAAGGAATATAAAATGAGA
GTAAGATCACATGGTAACAATATGTTTCTTAGTCATAATCAGATCTAACAAATCAAGCTA
ACATATATTTATAATTAAAAGTACTACATCAACTGGACTAAAAACTTGAGCCAAAAGCTT
TATAAAGAAAAAAGGATAATATAAAATTGAATAGTACGTAACCCAAAAGTCACTGGAGTT
AGAATTTGTGATATTGTTGTCCAAGCTTTGGATTTTTGATATCAGATGATTATATATAAA
AAAATGTAGTTATGCAAAAAATCAACAAATCATAAAAAAATAAATAAGCATATGACTTAT
ATATAAGTACTTACAAATGATATTAGTAAATACTTATTTACAGTTGATATTTATAGTTTT
ATACCAAATAAAATAATCATATCTTAAGCATCACAAGTTTAAAAAGTGAAAAGACATACT
ATATAATTATCATTTGACAATACTCAGTATTAATTAATTTTTATCTGTCGAAATTCACAT
GTAACCTAGTGTTAATGATAATTGCCCGATCATTAGTTAGCAGAGTTACGATGATAAGCG
ATAGGCACCCGATAAAACAATCAAGACATGCAAGTAGACCGAATACGATTCTACAAAATA
TTGACCGAATACATTACACGTTGGTGGTGTCGCCCTTGGACCCTGCTGAANNNNNNNNNN
NNNNNNNNNNNNNNNNNNNTGAACGCGAAATACTTTGTACACCAGACTATCTTTTAGTAT
CGACCGAATATGATACTAAAAATATCGACCGAATACATCACACCCTTGGTGGTACAACCC
TTTCCTAGACACTACATGAATGCGAGATACTTTGATTTGTGCACCGGACTACCTTTTAAT
GTCGATCAAACACGATACTAAAACGCAAGATGCTTTGTGCACCAGACTACCTTTTATTGT
CGATCGAATACGATACTAAAAATGTCAACCGAACACAGCACACCCTTGGTGGTACAACCC
TTTCCTAGACACTACATGAACGCGATATACTTTAATTTGTGCATCGGACTATCTTTTAGT
GTCGATCGAATACAATACTAAAACGCAAGATGCTTTGTGCACCAGACTACCTTTTATTGT
CGATCGAATACGATACTAAAAATGTCAACCGAACACATCACACCCTTGGTGGTGCAACCC
TTTCCTAGACACTACATGAATGCGATATACTTTAATTTGTGCACCGGACTACCTTTTAGT
GTCGATCGAACACGATACTAAAACACAAGATGCTTTGTGCATCAGACTACCTTTTATTGT
CGACCGAATAAGATACTAAAATGTCAACCGAATACATCACACCTTTGGTGGTACAACCCT
TTCCTGGATCCTGCATAAAAGCGAGATGCTTTTTACACCAGACTACTTATATATTATACG
ATACGAAAAATAATGTTAACCGAATACATCACACCCTTGGTGGTACAATCTTTTCCTAGA
TCTTCCATGAATGCGAGATGTTTTGTGCACCAGATTACCTTGTTGTATTGACCGGATACA
ACACTAATTTTTTTTTTTTTTTTTTGCACAACCTCCTTCGTGGTAGCTGCATATATATAT
AGTTAAAATAGACCAATAAGATCCAGAAATCTATAGTCAAACCATGTACATTATTTTATT
CATGTTCAATATTTCCCATATGAACAGCGTGCCTCACTATTGTTTCTTGACTTATCAATT
CTAAGTTCCACGAAAACGACCTTCACTAATTACTGCACAGTCCACCTTCTTATGCCGTCC
AATCTCACCATTAAATCATAGCTCCATTTCACCAATATTCTTTTGTAGCCAATTCTCCTA
AACAAATTCTTGAAAAATAGAAGAAACAAAAACATAATCCTCACTCTTCTCTCCTCAGCA
