# Supplementary material for: High Temperature Induces Expression of Tobacco Transcription Factor NtMYC2a to Regulate Nicotine and JA Biosynthesis
Source: Front Physiol. 2016 Oct 27;7:465. doi: 10.3389/fphys.2016.00465 (PMC5081390; doi:10.3389/fphys.2016.00465)
Supplement: Supplementary File 2 — The sequence information for the promoter of NtPMT1. The G-box motif were highlighted, and the F1, F2, and F3 fragment for CHIP assay were labeled by different color. [file Presentation3.pdf]

>NtPMT1 promoter sequence

GTATACCAAAAATCAATTCAACCCCCAAAACATAATACAACCAATGTTAATGCAATA
TCTCTGCTGCTATCACGAAGATAATTGTAGCTCACGAAAGTAGGATACATTATGTAGG
TTACATCACATAGAGGTAATCTAAAGCTCCCAATAATAAGATGTGTAATGTTGATTAT
GTAGAAATTTGCCAGGTTATTTAGAATAAACAAGAAGAGGAGAAAAAAAGTACAATT
TACCTGAACTCTTGAATGTATCCTACAAATAACCTAGACTTCATGG
ACGTCAGTTGTCAGTTTACTTTTGTTTTAATGGTACATCATTTGTCAAATACTTTATTT
GGATAAAAACAGTTTTGCCTAAGGAGTAAACAGATCCGGAGTAAGAAAGCAGACGA
TTAAAGCAATTTTTAAAAAAGGAGAGAGAAATTAATGAGCACACACATATACTAGTG
AAATTAGGGTACTAATTTACTAATAATTGCACCGAGACAAACTTATATTTTAGTTCCA
AAATGTCAGTCTAACCCTGCACGTTGTAATAAATTTTTAACTCTAT
TATATTATATCGAGTTGCGCCCTCCACTCCTCGGTGTCCAAATTGTATTTAAATGCATA
GATGTTTAATGGGAGTGTACAGCAAGCTTTCGGAAAATACAAACCATAATACTTTCTC
TTCTTCAATTTGTTTAGTTTAATTTTGAAAATGGAAGTCATATCTACCAACACAAATG
GCTCTACCATCTTCA
